# Secretome Analysis Defines the Major Role of SecDF in *Staphylococcus aureus* Virulence

**DOI:** 10.1371/journal.pone.0063513

**Published:** 2013-05-03

**Authors:** Chantal Quiblier, Kati Seidl, Bernd Roschitzki, Annelies S. Zinkernagel, Brigitte Berger-Bächi, Maria M. Senn

**Affiliations:** 1 Institute of Medical Microbiology, University of Zurich, Zurich, Switzerland; 2 Division of Infectious Diseases and Hospital Epidemiology, University Hospital Zurich, University of Zurich, Zurich, Switzerland; 3 Functional Genomics Center Zurich, Swiss Federal Institute of Technology and University of Zurich, Zurich, Switzerland; National Institutes of Health, United States of America

## Abstract

The Sec pathway plays a prominent role in protein export and membrane insertion, including the secretion of major bacterial virulence determinants. The accessory Sec constituent SecDF has been proposed to contribute to protein export. Deletion of *Staphylococcus aureus secDF* has previously been shown to reduce resistance, to alter cell separation, and to change the expression of certain virulence factors. To analyse the impact of the *secDF* deletion in *S. aureus* on protein secretion, a quantitative secretome analysis was performed. Numerous Sec signal containing proteins involved in virulence were found to be decreased in the supernatant of the *secDF* mutant. However, two Sec-dependent hydrolases were increased in comparison to the wild type, suggesting additional indirect, regulatory effects to occur upon deletion of *secDF*. Adhesion, invasion, and cytotoxicity of the *secDF* mutant were reduced in human umbilical vein endothelial cells. Virulence was significantly reduced using a *Galleria mellonella* insect model. Altogether, SecDF is a promising therapeutic target for controlling *S. aureus* infections.

## Introduction

The Gram-positive pathogen *Staphylococcus aureus* is one of the leading causes of nosocomial infections [1]. Due to its acquisition of various resistance genes treatment of *S. aureus* infections have become increasingly difficult. Furthermore, the prevalence of methicillin-resistant *S. aureus* (MRSA) has been increasing in recent years. This has resulted in an alarming rise of community-associated (CA-) MRSA infections in immunocompetent individuals [2], [3], [4]. In addition to its adaptive response to antibiotics [5], the success of *S. aureus* is based upon its huge array of virulence factors [6] helping *S. aureus* to avoid host immunity. These virulence factors have to be exported across the cytoplasmic membrane to reach their destined location: the membrane, the cell wall or the extracellular space. The main transport system is the Sec translocase, which is conserved in all three kingdoms of life [7], [8]. Currently, the Sec pathway is best described in the Gram-negative bacterium *Escherichia coli* (as reviewed in [9], [10]). The translocase consists of i) the heterotrimeric complex SecYEG, which forms a hydrophilic channel through the cytoplasmic membrane; ii) the motor protein SecA, an ATPase; and iii) the heterotrimeric complex SecDF-YajC. Proteins containing an N-terminal Sec signal peptide (SP) or a hydrophobic transmembrane segment are targeted to the translocase and transported through the channel in an unfolded state. For secreted proteins or membrane proteins with large hydrophilic loops, the driving energy is provided by the cycling of SecA, whereas ribosome-bound nascent chains are targeted mainly by inner membrane proteins [11] and are co-translationally exported powered by the translating ribosome. Small membrane proteins can also be inserted by YidC in a Sec-independent manner [12]. The auxiliary complex SecDF-YajC was shown to associate with SecYEG [13] as well as with YidC and is therefore believed to act as the linking molecule between SecYEG and YidC during Sec-dependent membrane protein insertion [12]. The integral membrane protein YajC was found to co-crystallize with the well-known *E. coli* multidrug exporter AcrB [14], which belongs to the resistance-nodulation-cell division (RND) superfamily. Deletion of YajC only showed a weak phenotype and its exact function is still unknown [15], [16].

SecDF also belongs to the RND superfamily and possesses the typical twelve transmembrane (TM) domains with two extracytoplasmic loops between TM1-2 and TM7-8, respectively [17]. Recently, Tsukazaki *et al.* resolved the crystal structure of the membrane protein SecDF of *Thermus thermophilus* [18]. Two conformations for the head subdomain P1 are observed, that seem to occur upon rotation by 120° [18]. Furthermore the P1 head was shown to interact with an unfolded preprotein, thereby preventing the substrate from backsliding and enhancing the translocation. This ATP-independent step in the later stage of protein translocation is driven by the proton motive force (PMF). Two conserved charged residues Asp519 and Arg247 are crucial for SecDF activity in *E. coli* and point mutations of the corresponding amino acids in *T. thermophilus* abolish ion channel activity [18].

In *S. aureus*, SecA and SecY have been shown to be essential [19], [20], [21]. Deletion of the non-essential *secG* leads to changes in the exoproteome, which are enhanced in a *secG*-*secY2* double mutant [7]. SecY2 together with SecA2 belong to the accessory Sec pathway, which at present is known to export only one substrate, the serine-rich adhesin for platelets protein (SraP) [22]. However, virulence of the *secG* and *secY2* single mutants and the *secG*-*secY2* double mutant in mice is comparable to the parental strain [7].

We previously reported a *S. aureus secDF* mutant to have a pleiotropic phenotype influencing not only protein secretion, but also transcription and regulatory processes [23]. Resistance towards β-lactam and glycopeptide antibiotics was reduced. Furthermore, cell division was impaired and autolysis increased [23]. To determine the role of SecDF in the secretion of virulence factors and to assess its importance for pathogenesis, we performed a secretome analysis using isobaric tags for relative and absolute quantitation (iTRAQ) with subsequent LC-MS/MS. Major virulence determinants involved in adhesion to host proteins and cells, as well as in evasion of the host immune system were found to be decreased in the exoproteome of the *secDF* mutant. Important steps for establishing an infection were shown to be deficient *in vitro* in the *secDF* mutant in both methicillin sensitive and resistant *S. aureus* strains. Furthermore, *secDF* virulence was significantly reduced in a *Galleria mellonella* infection model.

## Materials and Methods

### Bacterial strains and growth conditions

Bacterial strains and plasmids used in this study are listed in Table 1. If not mentioned otherwise bacterial cultures were grown in Luria Bertani (LB) broth (Becton Dickinson, Difco Laboratories, Franklin Lakes (NJ), USA) at 37 °C. Bacterial cultures were grown under constant shaking and with a liquid-to-air ratio of 1∶5 to assure good aeration. Media were supplemented with 50 µg/ml kanamycin (Sigma-Aldrich, St. Louis (MO), USA) or 10 µg/ml erythromycin (Sigma-Aldrich, St. Louis (MO), USA) when appropriate.

**Table 1:** Strains and plasmids used in this study.

Strains	Properties	Ref.
*S. aureus*		
CHE482	CC45, ST45, SCC*mec*_N1_, *blaZ* (pBla), Fa^r^, Mc^r^, Sx^r^, Tm^r^	[51], [53]
Cowan I	NCTC8530, septic arthritis isolate	ATCC12598
CQ66	Newman Δ*secDF*	[23]
CQ85	Newman pCN34, Km^r^	[23]
CQ87	Newman Δ*secDF* pCN34, Km^r^	[23]
CQ89	Newman Δ*secDF* pCQ27, Km^r^	[23]
CQ92	CHE482 pCN34, Km^r^	This study
CQ93	ME305 pCN34, Em^r^, Fa^r^, Km^r^, Mc^r^, Sx^r^, Tm^r^	This study
CQ94	ME305 pCQ27, Em^r^, Fa^r^, Km^r^, Mc^r^, Sx^r^, Tm^r^	This study
ME305	CHE482 Δ*secDF*::*bursa aurealis*, Em^r^, Fa^r^, Mc^r^, Sx^r^, Tm^r^	[54]
Newman	Clinical isolate (ATCC 25904), *rsbU*+	[83]
**Plasmids**		
pCN34	*S. aureus*-*E. coli* shuttle vector, pT181-*cop-wt repC aphA-3* ColE1, Km^r^, called pEmpty in this study	[84]
pCQ27	pCN34 derivative carrying *secDF* with its endogenous promoter, Km^r^, called pSecDF in this study	[23]

Abbreviations are as follows: Em^r^, erythromycin resistant; Fa^r^, fusidic acid resistant; Km^r^, kanamycin resistant; Mc^r^, methicillin resistant; Sx^r^, sulfomethoxazole resistant; Tm^r^, tobramycin resistant.

### Sample preparation for secretome analysis

Strains were cultured until late exponential phase, which was shown to correspond to OD_600_ 1 in an earlier study [23] and centrifuged at 4 °C for 5 min. The supernatant (SN) was filtered (0.22 µM PES filter, Techno Plastic Products AG, Trasadingen, Switzerland) and mini EDTA-free complete protease inhibitors tablets (Roche, Rotkreuz, Switzerland) were added. For normalization purposes 500 pM enhanced GFP (Ams Biotechnology Ltd, Abingdon, UK) was added. The SN was concentrated by trichloroacetic acid (TCA) precipitation and washed twice with ice cold (−20 °C) acetone. The pellet was resuspended in 0.5 M triethylammonium bicarbonate pH 8.5 (Sigma-Aldrich, St. Louis, USA) 0.5 mM tris(2-carboxyethyl)phosphine hydrochloride (TCEP, Sigma-Aldrich, St. Louis (MO), USA). Protein concentration was measured with the Quant-iT™ protein assay kit (Life Technologies, Invitrogen, Carlsbad (CA), USA).

Proteins were digested and labelled according to the iTRAQ protocol (AB Sciex, Concord, Canada). Briefly, 75 µg protein were denatured with 0.1% SDS and reduced with 5 mM TCEP. 10 mM methyl methanethiosulfonate (MMTS) was used as a cysteine blocking reagent. Protein samples were digested with trypsin (Promega, Fitchburg (WI), USA) for 14.5 h at 37 °C and subsequently labelled with a 4-plex-iTRAQ Reagent for 1.5 h at room temperature. Phosphoric acid was added to stop the reaction and samples were combined into a fresh Eppendorf tube. Peptides were fractioned by strong cation exchange (SCX). Solvent A (7 mM KH_2_PO_4_, 25% acetonitrile (ACN), pH 2.7) was added to the sample and loaded onto a polysulfoethyl A column (200×2.1 mm, 5 µm, 200 Å, PolyLC) of the analytical HPLC (LC1100, Agilent Technologies, Santa Clara (CA), USA). Peptides were eluted with an increasing gradient of solvent B (7 mM KH_2_PO_4_, 0.5 M KCl, 25% ACN, pH 2.7) 10–50 min, 0–30% solvent B; 40–60 min, 30–100% solvent B) and pooled into 12 fractions according to the chromatogram. Peptides were concentrated with the SpeedVac® (Eppendorf, Hamburg, Germany), redissolved in 3% ACN, 0.1% trifluoroacetic acid and desalted using ZipTips C_18_ (Merck Millipore, Billerica (MA), USA). After a further vacuum concentration step the peptides were dissolved in 3% ACN, 0.1% formic acid (FA).

### Mass spectrometry

Dissolved samples were injected into an Eksigent-nano-HPLC system (Eksigent Technologies, Dublin (CA), USA) by an auto sampler and separated on a self-made reverse-phase tip column (200 µm×150 mm) packed with C_18_ material (3 µm, 200 Å, AG, Bischoff GmbH, Leonberg, Germany). The column was equilibrated with 99% solvent A (1% ACN, 0.2% FA in water) and 1% solvent B (0.2% FA in ACN). Peptides were eluted using the following gradient: 0–3 min; 1–5% B, 3–57 min; 5–35% B, 57–63 min; 35–50% B and 63–70 min; 50–99% B, at a flow rate of 0.7 µl/min. High accuracy mass spectra were acquired with an AB Sciex 5600 (AB Sciex, Concord, Canada) in the mass range of 400–1250 m/z. Up to 40 data dependent MS/MS were recorded in high sensitivity mode of the most intense ions with charge state 2+, 3+ and 4+ using collision induced dissociation. Target ions already selected for MS/MS were dynamically excluded for 90 s after tree accuracies. After data collection, the peak lists were generated and analyzed using ProteinPilot™ 4.0 (AB Sciex, Concord, Canada). Data was searched against a SwissProt database (released January 2011). The following search parameters were used: Trypsin digestion, modifications of MMTS labelled cysteine, 4-plex-iTRAQ modifications of free amines at the N-termini and of lysine, and 4-plex-iTRAQ modifications of tyrosine. Biological modifications and single amino acid exchanges were also included in the search. No normalization for iTRAQ ratios, such as BIAS correction, was applied, as normalization was performed by the addition of extrinsic GFP. The peptides without any 4-plex-iTRAQ label at the N-terminus or at a lysine were excluded from the analysis. The ProteinPilot cut-off score used was 1.3, which corresponds to a confidence limit of 95%. In total four biological replicates were analyzed in two independent iTRAQ experiments. The statistical analysis was assessed by the Student's *t* test.

### SDS-PAGE and Western blot

The SN was collected using the secretome sample preparation method and concentrated with Amicon Ultra-15 centrifugal filter devices (MWCO 10 kDa Merck Millipore, Billerica (MA), USA). Ten to 20 µg of protein were separated by SDS (10–15%) polyacrylamide gel electrophoresis (PAGE) and blotted onto polyvinylidene fluoride (PVDF) membrane (Immobilon-P, Merck Millipore, Billerica (MA), USA). Blocking and detection were performed in phosphate buffered saline (PBS) pH 7.4 (CHIPS, FnBPA) or in low salt buffer (0.154 M NaCl, 9 mM TRIS, 0.1% Tween) pH 7.4 (Eap, LytM, SceD and SEA) as described in [23]. The following primary antibodies were used: Mouse anti-CHIPS antibody (Abcam, Cambridge, UK) 1∶1'000, rabbit anti-Eap antibody (Abcam, Cambridge, UK) 1∶5'000, rabbit anti-FnBPA antibody (Abnova, Taipei City, Taiwan) 1∶2'500, rabbit anti-LytM antibody (obtained from T. Msadek [24]) 1∶50'000, rabbit anti-SceD antibody (obtained from S. Foster [25]) 1∶10'000 and rabbit anti-SEA antibody (Abcam, Cambridge, UK) 1∶1'000. For detection of the primary antibodies either horseradish peroxidase-(HRP-) goat anti-rabbit IgG (1∶10'000, Jackson ImmunoResearch, West Grove (PA), USA) or HRP-goat anti-mouse IgG (1∶5'000, Jackson ImmunoResearch, West Grove (PA), USA) were used.

### Fibrinogen- and fibronectin-binding assay

The binding assay was adapted from O'Neill *et al.* [26]. Shortly, 100 µl two-fold dilutions of human fibrinogen (Merck, Calbiochem, Darmstadt, Germany) in 20 mM sodium citrate-HCl, pH 7.4 or human fibronectin (Merck, Calbiochem, Darmstadt, Germany) in PBS pH 7.4 were dispensed in flat-bottom 96-well Nunclon plates and incubated overnight at 4 °C. Following three PBS wash steps, the plates were blocked in 2 mg/ml bovine serum albumin (BSA, Thermo Scientific, Acros Organics, Geel, Belgium) in PBS for 2 h at 37 °C. Wells were washed three times in PBS. In the meantime bacterial cultures were grown to OD_600_ 1, washed twice in PBS and adjusted to OD_600_ 1 in PBS corresponding to ∼10^8^ cells/ml. Hundred µl of bacterial suspension was added per well (10^7^ CFU/well), including a negative control (PBS) and incubated for 2 h at 37 °C. After repeated PBS washing steps, the cells were fixed with 25% formaldehyde (Applichem, Darmstadt, Germany), washed once with PBS and stained for 5 min in 0.5% crystal violet. Residual dye was removed with distilled water (dH_2_O) and plates were air-dried. The crystal violet stained cells were dissolved in 5% acetic acid and the absorbance measured at 570 nm. The experiment was performed with three technical and biological replicates, except for the positive control strain Cowan I in the Newman fibronectin binding assay, which was only performed with three technical replicates.

### Tissue culture

Human umbilical vein endothelial cells (HUVECs) were purchased from Clonetics (Lonza, Basel, Switzerland) and maintained as previously described in M199 medium supplemented with antibiotics (penicillin 100 U/ml, streptomycin 100 µg/ml), glutamine at 2 mM, and 20% foetal calf serum, at 37 °C in a humidified 5% CO_2_ atmosphere until they reached confluency [27]. One day before assays were performed, HUVECs were seeded at a density of 5×10^4^ cells/well into 48-well plates that were coated with 10 µg/ml fibronectin. Cells were used up to passage three.

### Bacterial adherence and invasion to HUVECs

The capacity of the various strains to invade HUVECs was determined by the lysostaphin protection assay using conditions used for the MTT assays (see below) as previously described [28]. Adherence and invasion were expressed as % adherent and invading cells per well at the time point of measurement. Adherence and invasion of CHE482 were set to 100% for each run. Each experiment was performed in duplicate in three independent assays.

### MTT assay

The ability of *S. aureus* to induce damage in HUVECs was assessed using an MTT (3-(4,5-dimethylthiazol-2-yl)-2,5-diphenyltetrazolium bromide) assay. MTT is reduced by living cells to insoluble purple MTT formazan crystals using succinate, and the pyridine nucleotide cofactors, NADH and NADPH as substrates [29]. MTT production is therefore inversely related to cell death. MTT reduction results in a yellow to blue color change that can be quantified by measuring the absorbance at OD_570_ [30]. MTT assays were performed as previously described with some modifications [31]. Briefly, wells containing HUVECs were rinsed twice with warm HBSS prior inoculation to remove medium containing antibiotics. The bacterial strains were grown overnight on sheep blood agar, resuspended in invasion medium (1% albumin and 25 mM HEPES, pH 7.3 in M199 without serum or antibiotics) to McFarland 0.5 (∼5×10^7^ cells/ml), sonicated and diluted to the indicated multiplicity of infection (MOIs) [32], [33], [34]. Initial inocula were confirmed by serial fold dilution and plating on sheep blood agar. After 1 h or 3 h incubation at 37 °C in 5% CO_2_, respectively, the number of CFUs per well was determined to monitor growth. Then, the medium was aspirated, wells were washed with HBSS and 400 µl of fresh complete M199 medium containing 10 µg/ml lysostaphin was added [28], [33], [35]. After a total incubation time of 22 h at 37 °C in 5% CO_2_, 100 µl of 5 mg/ml MTT in HBSS were added to each well. 2 h later, the medium was removed and 150 µl of 0.04 M HCl in absolute isopropanol were added to solubilize the dye. Uninfected control wells which underwent the same washes were processed in parallel and served as negative control. Wells that only contained medium were used as background correction. Absorbance of 100 µl of the solution was measured at 570 nm. Specific cytotoxicity was calculated using the following formula: 1-(OD_570_ experimental well/OD_570_ control well). Each experiment was performed in triplicate in at least three independent assays.

### Galleria mellonella virulence assay

To study the *in vivo* pathogenicity an invertebrate infection model was used as previously described by Peleg *et al.* [36]. Last instar larval stage *G. mellonella* were purchased at HRH Fishing Hebeisen, Zurich, Switzerland and stored at 4 °C until further use. Cells were grown until exponential phase (OD_600_ 0.5), washed twice in PBS and resuspended therein. Ten µl of bacterial suspension, corresponding to 10^6^ CFUs, were injected into the last left proleg with a repetitive dispensing Tridak Stepper (Intertronic, Oxfordshire, UK) containing a 1 ml syringe with a 26-gauge needle. Thirty larvae were infected per strain. A control group was inoculated with 10 µl of PBS to assure the larvae were healthy and that death did not occur due to the needle prick or stress. Larvae were incubated at 37 °C and examined every 24 h for survival; they were considered dead when no movement occurred in response to touch. Additional larvae were inoculated separately to measure the bacterial burden which was monitored after 24, 48 and 72 hours. After incubating the larvae at −20°C, they were disinfected in 70% ethanol and rinsed with dH_2_O. The larvae were homogenized with the TissueLyser (Qiagen, Hombrechtikon, Switzerland) in 2-ml screw cap tubes containing a 5 mm stainless steel bead (Qiagen, Hombrechtikon, Switzerland) and 1 ml PBS, shaking for 10 min at 30 s^−1^. Appropriate dilutions were plated on LB agar containing 50 µg/ml kanamycin to minimize growth of the normal flora of the larvae. Three independent experiments were pooled and analysed by log rank test. The survival-curves were plotted using the Kaplan-Meier method.

## Results

### Deletion of *secDF* leads to an altered exoproteome

To determine the extent of the extracellular proteome affected by deletion of *secDF*, a quantitative secretome analysis was performed for the *S. aureus* wild type strain Newman and its mutant strain Newman Δ*secDF* using iTRAQ. The SNs were collected during late exponential phase, where we found strongest SecDF expression (data not shown). Samples of four biological replicates were prepared for LC-MS/MS as described in materials and methods.

**Figure 1:**
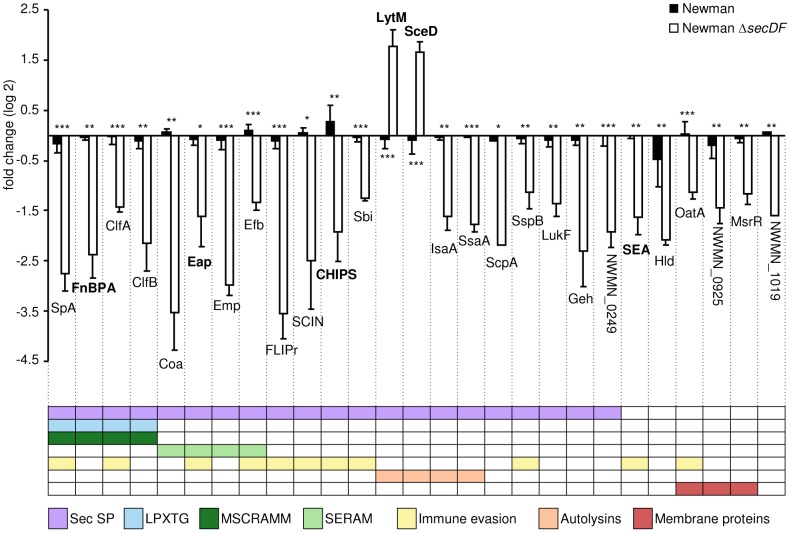
Differential extracellular protein amounts in Newman Δ*secDF* in comparison to the wild type Newman. Proteins identified to be significantly and more than two-fold changed in Newman Δ*secDF* compared to the wild type Newman. The mean values of four independent experiments are shown with their standard deviation given, except for NWMN_1019 and ScpA, which were only found in two biological replicates. Proteins are colour coded according to the following categories: N-terminal Sec signal peptide (SP), LPXTG-motif, adhesive properties (MSCRAMM and SERAM), immune evasive properties, autolytic properties and membrane proteins. Proteins confirmed below by Western blot analysis are highlighted in bold. *, *P*<0.05; **, *P*<0.01; ***, *P*<0.001.

A total of 230 *S. aureus* proteins were quantified and their putative localization was determined with different bioinformatics tools (Table 2, Table S1). Thirty-eight proteins had a predicted SP [37], 34 thereof were predicted Sec-type SPs [38]. These included seven cell wall proteins containing the LPXTG cell wall retention motif (SpA, SdrE, ClfA, IsdA, FnBPA, ClfB) [39], [40] or LysM domains (Sle1/Aaa) [41]. In Newman Δ*secDF*, in comparison to the wild type Newman, the extracellular levels of 27 proteins were altered significantly by at least two fold increase (two proteins) or two fold decrease (25 proteins); 21 of these proteins contained a Sec-type SP (Figure 1). Of the six remaining proteins, three were membrane proteins; OatA and the LytR-CpsA-Psr proteins NWMN_0925 (SA0908) and MsrR [42], [43]. Furthermore, the SP containing, but Sec-independent enterotoxin A (SEA) and the delta hemolysin (Hld) were identified.

**Table 2:** Localization of identified proteins based on different bioinformatics tools [85], [86], [87] and Sibbald *et al.* [38].

Localization	Proteins (%)	Sec signal peptide	Remarks
Cytoplasm	172 (74.8)		
Membrane	9 (3.9)		
Lipoprotein	2 (0.9)		
Cell wall	7 (3.0)	7	6 covalently attached by LPXTG motif
Extracellular	31 (13.5)	27	6 non-covalently bound to the cell wall
Unknown	9 (3.9)		
Total	230 (100)		

According to their function the majority of the proteins were classified into three groups: i) Proteins with adhesive properties (ClfA, ClfB, Coa, Emp, Efb/Fib, FnBPA, Eap/Map, SpA) [44], [45]; ii) proteins which are involved in immune evasion (CHIPS, ClfA, Efb, FLIPr, Eap, OatA, Sbi, SpA, SCIN, SEA, SspB) [46], [47] and iii) autolytic proteins (IsaA, LytM, SceD, SsaA) [25], [48], [49]. The autolysin LytM and the lytic transglycosylase SceD were the only proteins found to be significantly increased in the secretome of Newman Δ*secDF*. Furthermore, several proteases were decreased in Newman Δ*secDF*, such as SspB, SspA and ScpA, whereof the amounts of SspB and ScpA were significantly reduced.

Peptides of the N-terminal region of the glycerol ester hydrolase Geh were identified and found to be reduced in Newman Δ*secDF*. Because one of the four prophages in strain Newman is located in the Geh gene *geh*, leading to a truncated and inactive enzyme [50], this finding is not phenotypically relevant in the strain Newman background. Nonetheless, in other *S. aureus* strain backgrounds, deletion of *secDF* can be expected to reduce lipase activity.

### Complementation of the altered virulence factor expression in the mutant strain Newman Δ*secDF* and verification of its phenotype in a second strain background

To validate and further highlight the role of SecDF in *S. aureus*, a different, methicillin resistant strain background was included in the following confirmatory experiments. The low level methicillin resistant strain CHE482 represents a CA-MRSA [51], [52], [53]; its corresponding *secDF* mutant CHE482 Δ*secDF* carries a transposon at position 1029 bp leading to a non-functional SecDF protein [54]. To complement the deletion of *secDF*, the plasmid pSecDF (pCQ27) containing *secDF* with its endogenous promoter from strain Newman was introduced into the mutant strains Newman Δ*secDF* and CHE482 Δ*secDF*, yielding Newman Δ*secDF* pSecDF and CHE482 Δ*secDF* pSecDF, respectively. The empty vector pEmpty (pCN34) was introduced into the wild type strains Newman and CHE482 and the *secDF* mutants Newman Δ*secDF* and CHE482 Δ*secDF* to ensure no additional effects were caused by the plasmid (Table 1).

To confirm the secretomics results, Western blot analysis of the SN from these strains were performed for selected proteins found to be altered in the secretome of Newman Δ*secDF*. Specific antibodies were used for the Sec-dependent proteins CHIPS, Eap, FnBPA, LytM and SceD, as well as the Sec-independent SEA.

**Figure 2:**
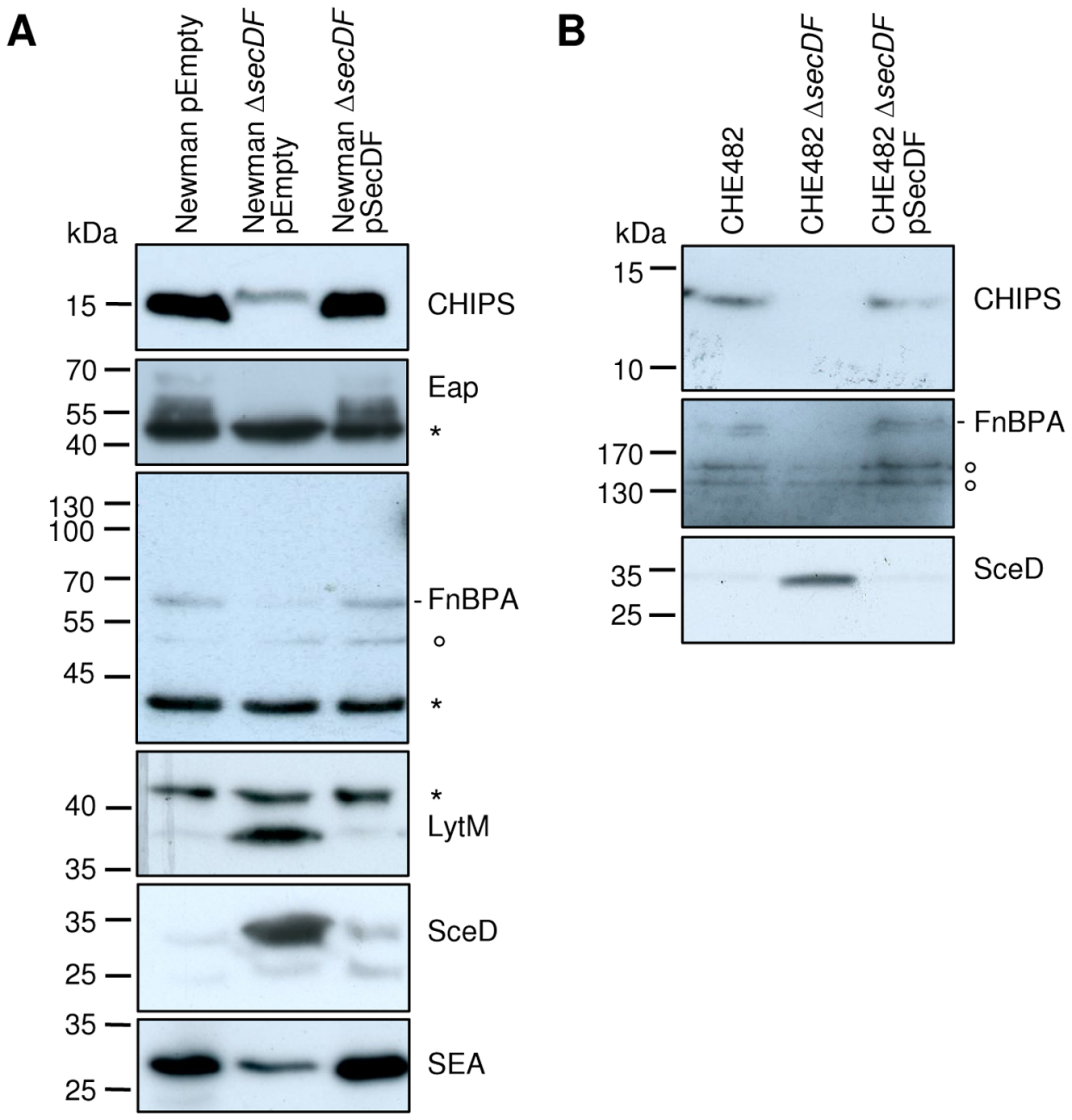
Complementation of SecDF-dependent changes. Western blot analysis of extracellular proteins in the SN of two different strain backgrounds. (A) Newman harbouring empty plasmid pEmpty (pCN34), the mutant strain Newman Δ*secDF* harbouring empty plasmid pEmpty (pCN34) and the complemented mutant Newman Δ*secDF* mutant harbouring plasmid pSecDF (pCQ27) containing the *secDF* gene with its endogenous promoter. (B) CHE482, the mutant strain CHE482 Δ*secDF* and the complemented mutant strain CHE482 Δ*secDF* harbouring plasmid pSecDF (pCQ27). Specific antibodies against CHIPS, FnBPA, Eap, LytM, SceD and SEA were used. Truncated FnBPA in Newman runs below the 70 kDa marker band. Putative degradation bands of FnBPA have been observed before [56], [80], [81], [82] and are indicated by a ring. Additional protein bands due to unspecific binding to protein A or Sbi are indicated by an asterisk.

As expected, the SN of Newman Δ*secDF* showed reduced protein amounts of CHIPS, FnBPA, Eap and SEA and increased amounts of LytM and SceD in comparison to the wild type and the complemented mutant (Figure 2A). This phenotype was validated and confirmed for CHIPS, FnBPA and SceD in strain background CHE482 (Figure 2B).

### Decreased adherence of the *secDF* mutant to attached human fibrinogen, fibronectin and endothelial cells

To establish an infection, the ability of *S. aureus* to adhere to host proteins and cells is essential and permits the bacteria to invade into the cells as shown previously for *S. aureus* [55]. Our secretomics screen revealed several factors belonging to the microbial surface components recognizing adhesive matrix molecules (MSCRAMM) or the secretable expanded repertoire adhesive molecules (SERAM) to be reduced in Newman Δ*secDF* (Table 3). Therefore, binding of the *secDF* mutants to immobilized human fibrinogen and fibronectin, respectively, was studied *in vitro*. Ninety-six-well-plates were coated with various concentrations of human fibrinogen and fibronectin, respectively. Bacteria were allowed to adhere to the coated wells, washed, fixed and coloured with crystal violet as described in materials and methods.

**Table 3:** Adhesins, which were reduced in the SN of Newman Δ*secDF*.

	Proteins	Binding to human fibrinogen	Binding to human fibronectin	Ref.
MSCRAMM	ClfA	x		[88]
	ClfB	x		[89]
	FnBPA	x	x	[80], [90]
	SpA			
SERAM	Coa	x		[91]
	Eap	x	x	[92]
	Emp	x	x	[93]
	Efb	x		[94]

**Figure 3:**
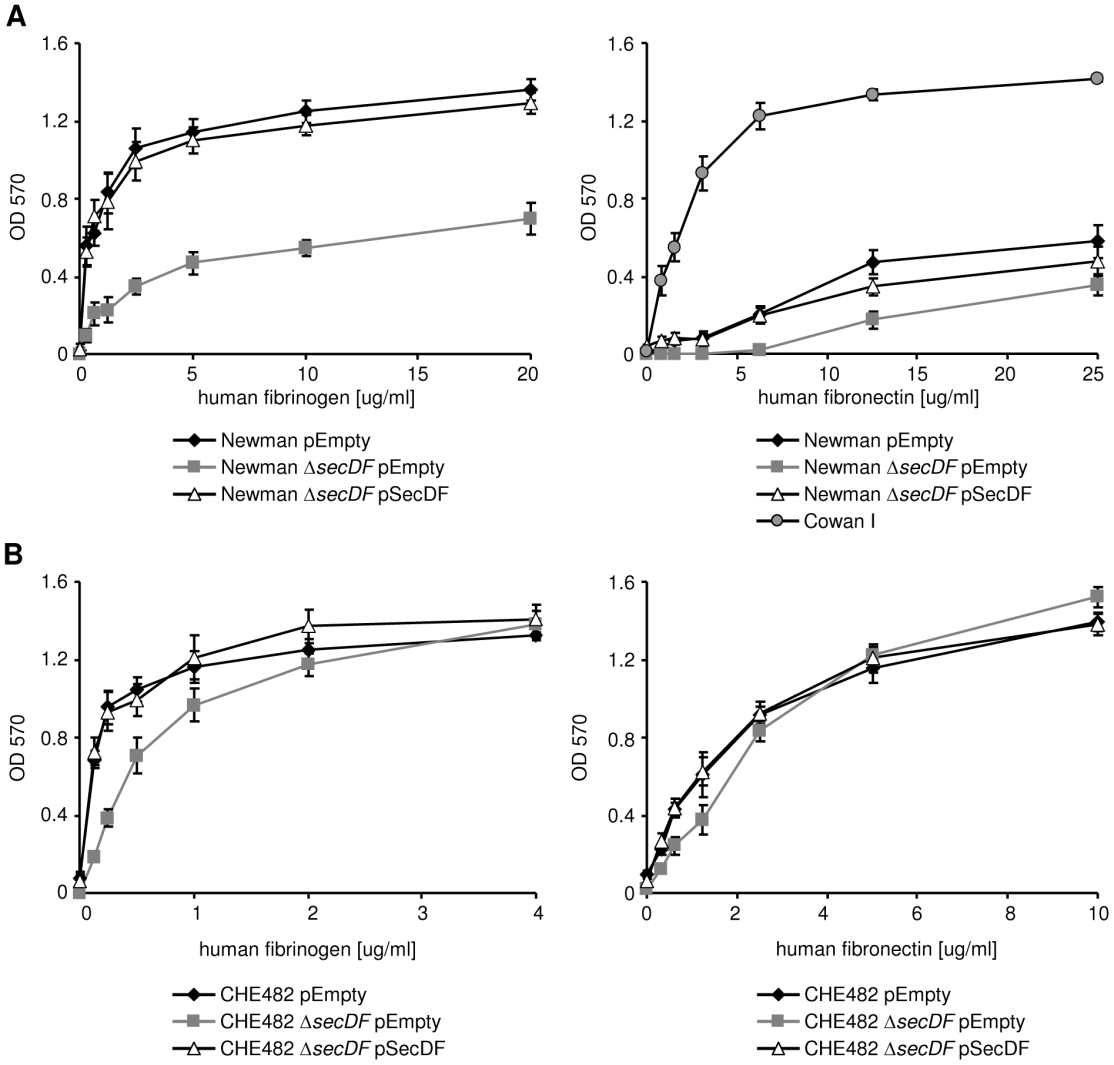
Impact of SecDF on fibrinogen and fibronectin binding. Binding properties of Newman and CHE482 strain sets to immobilized human fibrinogen and fibronectin was assessed as described in materials and methods. (A) Newman pEmpty, Newman Δ*secDF* pEmpty and the complemented mutant Newman Δ*secDF* pSecDF. FnBPs in the Newman background are truncated due to a point mutation leading to a stop codon before the sortase motif, which is required for cell wall anchoring, and therefore are secreted [56]. Hence, Cowan I was used as a functional control strain in the fibronectin binding assay. (B) CHE482 harbouring empty plasmid pEmpty (pCN34), the mutant strain CHE482 Δ*secDF* harbouring empty plasmid pEmpty (pCN34) and the complemented mutant CHE482 Δ*secDF* pSecDF. Mean of three independent experiments are shown with their standard deviation.

Binding of Newman Δ*secDF* pEmpty to fibrinogen was reduced by up to 50% in comparison to the wild type strain Newman pEmpty and the complemented mutant Newman Δ*secDF* pSecDF (Figure 3A). In CHE482, binding of CHE482 Δ*secDF* pEmpty was reduced at low concentrations of fibrinogen, but not at concentrations above 2 µg/ml (Figure 3B). This phenotype was restored in both strains by the complementing plasmid pSecDF.

Fibronectin binding proteins are not anchored to the cell wall in strain Newman due to a point mutation leading to an early stop codon and truncated proteins without sortase motif [56]. Therefore, strain Cowan I was used as a functional control in the binding assay of Newman to human fibronectin [56]. As expected, the binding capacity of strain Newman pEmpty to fibronectin was up to ten times lower as compared to strain Cowan I (Figure 3A). However, deletion of *secDF* further reduced binding to fibronectin in the Newman background and was partially restored by the complementing plasmid. In CHE482, the *secDF* mutant showed a similar concentration dependent phenotype for fibronectin as for fibrinogen, with the CHE482 Δ*secDF* pEmpty displaying a reduced binding at lower fibronectin concentrations (Figure 3B). These findings suggest the presence of additional fibrinogen and fibronectin binding proteins in the CHE482 background, possibly on mobile genetic elements, which reduce the effect of a *secDF* deletion on adhesion to fibrinogen and fibronectin.

**Figure 4:**
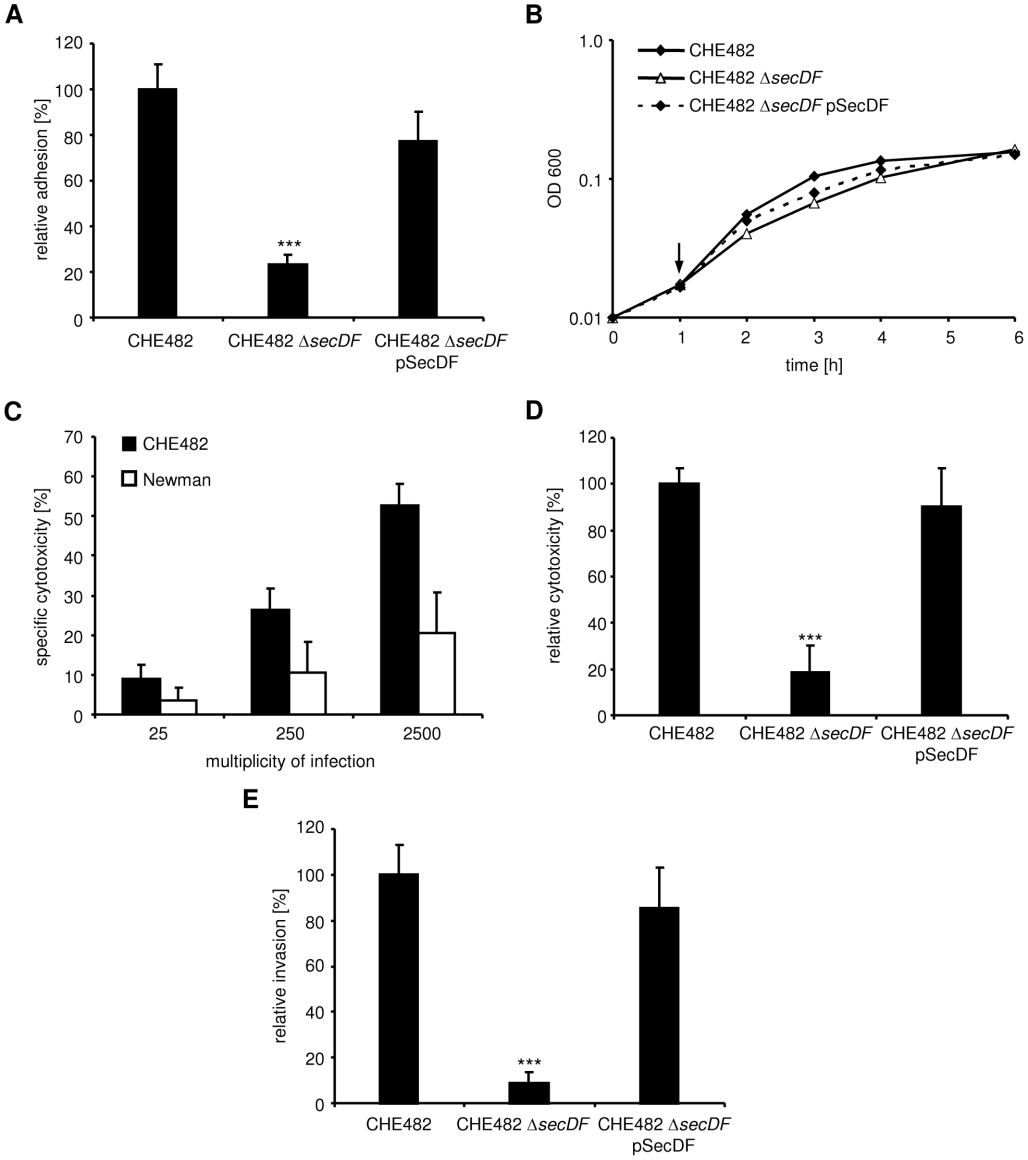
Adhesion, cytotoxicity and invasion in HUVECs. Interactions of Newman and the CHE482 strain set the wild type CHE482, CHE482 Δ*secDF* and the complemented mutant CHE482 Δ*secDF* pSecDF with HUVECs. (A) Effect of *secDF* inactivation in the CHE482 background on adhesion. (B) Growth in invasion medium. The arrow indicates the time point at which extracellular bacteria are lysed. (C) Inoculum dependent cytotoxicity of wild-type strains as determined by the MTT assay. (D) Effect of *secDF* inactivation in the CHE482 background on cytotoxicity as determined by the MTT assay. (E) Effect of *secDF* inactivation in the CHE482 background on invasion. ***, *P*<0.0001.

In a next step, we investigated whether the *secDF* deletion would also affect adherence to host endothelial cells (human umbilical vein endothelial cells, HUVECs). Because of the point mutation in the FnBPs of strain Newman mentioned above, this strain has been previously shown to be weakly adherent to HUVECs [56]. Therefore, we used the *secDF* mutant of strain CHE482 to test adherence and found significantly reduced adherence as compared to the wild type strain (Figure 4A). This effect was restored in the complemented mutant CHE482 Δ*secDF* pSecDF. In accordance with the previous findings [56] adherence by strain Newman was only 12.7±6.6% of strain CH482.

### *secDF* inactivation leads to reduced cytotoxicity

Many of the virulence factors produced by *S. aureus* destroy host tissue to allow dissemination. Thus, we analyzed the ability of the *secDF* mutant to damage endothelial cells using a previously published MTT assay [31]. This assay measures MTT reduction by living cells and is inversely related to cell death. We previously showed that MTT reduction by HUVECs internalized *S. aureus* is negligible [31]. To evaluate cytotoxicity of strain Newman, we used similar conditions as previously described using a MOI of 50 [33]. In this assay, endothelial cells are infected with bacteria for 3 h. Then, extracellular bacteria are killed and damage is assessed 24 h after the addition of the bacteria. Strain Newman did not induce substantial damage under these conditions (15.8±10.9% damage) and was not suited to study the effect of *secDF* inactivation on *S. aureus* cytotoxicity. We therefore decided to use strain CHE482, which caused a significant higher damage (69±15%) and the corresponding *secDF* mutant. However, CHE482 Δ*secDF* exhibited a growth defect in invasion medium during the 3 h invasion step (Figure 4B). We therefore decreased the invasion time to 1 h, while bacteria appeared to be in the lag-phase showing no measurable difference in OD_600_ or CFUs between the strains (Figure 4B). Because these conditions have not been previously used to study *S. aureus*-induced endothelial cell damage, it was essential to identify an optimal MOI. Figure 4C shows the increasing cytotoxicity of strain CHE482 with increasing MOIs ranging from 25 to 2500. Again, damage induced by strain Newman under these conditions was very low. Using an MOI of 2500, we found that *secDF* inactivation in strain CHE482 led to a substantial reduction in cytotoxicity (Figure 4D). This phenotype could be complemented using strain CHE482 Δ*secDF* pSecDF, which contains the *secDF* locus on a plasmid. To determine whether the reduced cytotoxicity of CHE482 Δ*secDF* was due to a reduction in endothelial cell invasion, invasion was assessed under similar conditions as for the MTT assay. The mutant strain CHE482 Δ*secDF* exhibited significantly reduced invasion as compared to the wild type strain (Figure 4E). This effect was restored in the complemented mutant CHE482 Δ*secDF* pSecDF. In accordance with the low adherence and cytotoxicity of strain Newman, invasion was only 1.6±1.0% of strain CHE482.

### Inactivation of *secDF* leads to reduced pathogenicity in an insect infection model

**Figure 5:**
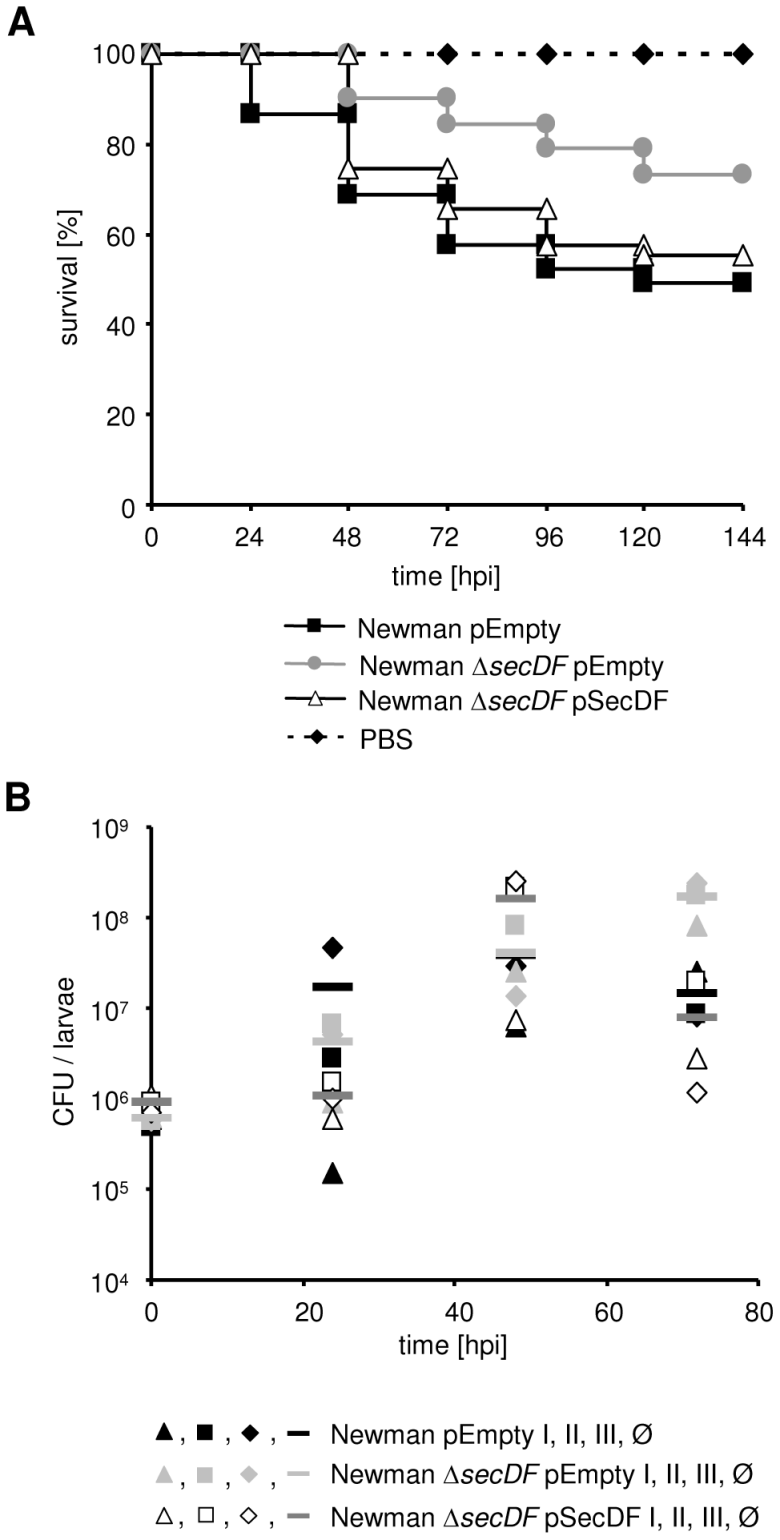
*G.* *mellonella* virulence assay. (A) Pathogenicity of Newman pEmpty, Newman Δ*secDF* pEmpty and Newman Δ*secDF* pSecDF in *G. mellonella*. Larvae were monitored every 24 hours. PBS was used as negative control. Three independent experiments were pooled and plotted as Kaplan-Meier survival curve, *P*<0.001. (B) Bacterial burden per (live) larvae was measured in triplicates 24, 48 and 72 h post infection (hpi). The symbols triangle, square, diamond and line correspond to replicates I, II, III and the average, Ø, respectively. The inoculum (CFU/larvae) is shown at time point zero.

To assess the influence of SecDF on virulence, Newman strains were injected into last instar larval stage *G. mellonella* and survival of the larvae was monitored over time. Resuspension buffer for bacterial cultures was used as a negative control. The dimensions and masses of larvae in one group spanned representative and comparable ranges, as preparatory experiments did not show any correlation concerning larval weight and survival (data not shown). Pathogenicity of Newman Δ*secDF* pEmpty in *G. mellonella* was significantly reduced (*P*<0.001) in comparison to the wild type Newman pEmpty and the complemented mutant Newman Δ*secDF* pSecDF (Figure 5A). To ensure that the attenuated virulence was not due to growth deficiencies of the mutant, the bacterial burden per larvae was measured after 24, 48 and 72 hours. Variation of CFU per larvae was rather high within the strains, for instance at 24 hours post infection (hpi) the wild type showed a bacterial burden ranging from 1.54×10^5^ to 4.62×10^7^ (Figure 5B). However, all three strains were able to multiply in the larvae within a similar range during the first 48 hpi, with CFUs being even higher in the *secDF* mutant after 72 hpi than in the wild type and the complemented mutant. The different peak time points of CFU/larvae in the *secDF* mutant (72 hpi) compared to the wild type or complemented mutant (48 hpi) was not reflected in the survival of *G. mellonella*, further indicating a strongly reduced virulence in Newman Δ*secDF* pEmpty, that was not compensated by increased CFU numbers.

## Discussion

Secretion of numerous virulence factors such as adhesins, proteases, autolysins and toxins rely on a functional Sec secretion system for the export across the cytoplasmic membrane [38]. So far, most studies of the Sec machinery were performed in the Gram-negative and Gram-positive model organisms *E. coli* and *B. subtilis*, respectively. This study focused on the impact of the auxiliary SecDF protein on virulence in the human pathogen *S. aureus* by analysing the exoproteome of Newman Δ*secDF* in a gel-free approach, followed by *in vitro* and *in vivo* virulence studies.

Our secretomics results were in good agreement with previous data [23]. A relatively high number of cytoplasmic proteins was found in the SN, which has already been observed by other research groups and in different microorganisms [57], [58], [59], [60]. This occurrence has been assigned to cell wall turnover, proteolytic processing, shedding, natural lysis and lysis due to handling [61], [62], [63]. Nevertheless, we identified several new proteins to be influenced by SecDF. This was confirmed by Western blot analysis and could be complemented for the proteins CHIPS, Eap, FnBPA, LytM and SEA.

Eight out of 11 proteins, identified in both the previously characterized *S. aureus* RN4220 *secG* mutant [7] and Newman Δ*secDF*, showed a similar trend and were found to be reduced in both mutants in comparison to the wild types (Sle1, Geh, Hlb, Hla, HlgB, HlgC, NWMN_1927 and YfnI). Three proteins (IsaA, Spa and SsaA) were found to be increased in the *secG* mutant, but reduced in Newman Δ*secDF*. Different methods, sampling time points and strain backgrounds could have contributed to those divergent findings. Another possibility is that SecDF is required for a subset of proteins deviating from the ones that require SecG. In addition, since they have different functions in the Sec pathway, the absence of SecG or SecDF might lead to only partially overlapping phenotypes.

Two Sec-dependent proteins were found to be increased in Newman Δ*secDF*; the autolysin LytM and the transglycosylase SceD. Kouwen *et al.* had observed, that a *B. subtilis* LipA hyper-producing strain could export the normally Sec-dependent LipA, which also contains a potential Tat RR-SP via both Tat pathways [64]. *S. aureus* LytM and SceD do not contain a potential Tat RR-SP; whether they are secreted by another transport system in Newman Δ*secDF* remains to be determined.

In the *secG* mutant only one Sec-independent protein was identified to be reduced; YfnI/LtaS [7], which was also found reduced in Newman Δ*secDF* (Table S1). Additionally, the two Sec-independent proteins Hld and SEA were found to be significantly reduced upon deletion of *secDF*. Hld belongs to the phenol-soluble modulins and has recently been shown to be exported by the ABC transporter Pmt [65]. The fact that the amounts of certain Sec-independent proteins were changed in both the *secG* and the *secDF* mutant, points towards indirect effects caused by the deletion of these Sec translocase constituents. Slightly reduced levels for the regulatory and Hld-encoding mRNA RNAIII have been shown previously in Newman Δ*secDF* [23]. Because RNAIII transcription is regulated by the two-component system accessory gene regulator (*agr*) the reduction of RNAIII levels suggest that *agr* is indirectly affected by *secDF* deletion [23], [66]. Clearly, more work is required to confirm this hypothesis and to determine whether other regulatory processes are influenced directly or indirectly by the absence of SecDF.

Secretome analysis showed decreased SpA levels in Newman Δ*secDF* consistent with a previously reported reduction of *spa* transcription [23]. In contrast, previous and present Western blot analysis showed similar levels of full-length SpA in the wild type and Newman Δ*secDF*. A possible explanation for these seemingly contradictory findings could be the reduced amounts of SspA protease in the Newman Δ*secDF* (Table S1). SspA is the main player of SpA degradation [67]. Higher SpA and SspA levels in the wild type would lead to more SpA degradation fragments, which can be detected by secretome analysis and increase the total protein amounts attributed to SpA, but would be too small to be detected by Western blot analysis. Whether this is the case still needs to be investigated.

Several well known virulence factors playing a central role during the initial steps of an infection were decreased in Newman Δ*secDF*. These changes led to reduced binding to the two important human host factors fibrinogen and fibronectin *in vitro*. Although the effect of the *secDF* deletion on the adhesion intensity varied between the two strains tested and was smaller in CHE482, we also found significantly reduced adhesion of CHE482 Δ*secDF* to HUVECs. This was accompanied by a significantly decreased invasion and must be attributed to the sum of impaired factors in the mutant and the multitude of additional host matrix factors *S. aureus* can bind to. In this study, the two important invasion factors Eap and FnBPA were found to be reduced [68], [69]. While the receptor of Eap is still unknown, the FnBPs bind via the bridging factor fibronectin to the host receptor integrins α_5_β_1_, which is sufficient to induce the uptake of staphylococci [68]. Thus, the low cytotoxicity in HUVECs displayed by CHE482 Δ*secDF* must be assumed to be at least partially caused by its defective adhesion and invasion, in addition to a reduced production of toxins. The decreased levels of numerous virulence factors and the reduced cytotoxicity, indicative for virulence in mice and rabbits [33], [70], [71], suggested the virulence of the *secDF* mutant to be attenuated. We therefore determined the pathogenicity of Newman Δ*secDF* pEmpty in the invertebrate model host *G. mellonella*, sharing several features of the innate immune response with mammalians [72], [73]. A positive correlation has been shown between the pathogenicity of microorganisms in insects and in mice [74], [75] and in recent years this model has been increasingly used for assessing the virulence of *S. aureus* [36], [76], [77], [78], [79]. As expected, virulence was significantly attenuated in Newman Δ*secDF* pEmtpy. The similar bacterial load per larvae indicated that Newman Δ*secDF* pEmtpy has the ability to multiply as well as the parent strain and that the generally diminished virulence factor expression Newman Δ*secDF* pEmtpy is the cause for its reduced virulence.

Taken together, our data provide new insights on the relevance of SecDF in *S. aureus* pathogenicity. We showed that deletion of *secDF* affects an important part of the extracellular proteome leading to reduced adhesion, invasion and cytotoxicity, as well as reduced virulence in *G. mellonella*. Because both MSSA and MRSA *secDF* mutants are less resistant to well established antibiotics [23], SecDF is an interesting target for the development of novel antimicrobial substances.

## Supporting Information

Table S1 Proteins found in the supernatant of *S. aureus* Newman and Newman Δ*secDF* and their predicted localization.
(PDF)
