# Supplementary material for: Secretome Analysis Defines the Major Role of SecDF in Staphylococcus aureus Virulence
Source: PLoS One. 2013 May 3;8(5):e63513. doi: 10.1371/journal.pone.0063513 (PMC3643904; doi:10.1371/journal.pone.0063513)
Supplement: Table S1 — Proteins found in the supernatant of S. aureus Newman and Newman ΔsecDF and their predicted localization. (PDF) [file pone.0063513.s001.pdf]

Supplemental Table S1. Proteins found in the supernatant of *S. aureus* Newman (wt) and the mutant Newman Δ*secDF* (*secDF*) and their predicted localization.

NWMN	Protein name	Gene	wt I	wt II	wt III	wt IV	*secDF* I	*secDF* II	*secDF* III	*secDF* IV	ø wt	ø *secDF*	*P*	SignalP	Sec SP	No SP(x)	SecretomeP	Localization [4,5]	
			fold change (log 2)											[1]	[2]	[2]	[3]		
0002	DNA polymerase III subunit beta	*dnaN*	0	0.16			0.01	-0.13			0.08	-0.06						Cytoplasm	
0014	50S ribosomal protein L9	*rplI*	0	-0.51			-0.61	-0.95			-0.26	-0.78					Yes	Cytoplasm	
0030	Probable tRNA-dihydrouridine synthase	*dus*	0	-0.12			-0.99	-0.32			-0.06	-0.66						Cytoplasm	
0055	Immunoglobulin G-binding protein A	*spa*	0	-0.37	0	-0.29	-3.01	-3.10	-2.40	-2.52	-0.16	-2.76	***	Yes	Yes		Yes	Cell wall	Sortase substrate
0113	Putative aldehyde dehydrogenase	*aldA*	0	0.16	0	-0.24	-0.15	-0.78	-0.46	-0.56	-0.02	-0.49	*					Cytoplasm	
0129	Ornithine aminotransferase 2	*argD*	0	0.05	0	-0.56	-0.34	-0.44	-0.68	-1.10	-0.13	-0.64						Cytoplasm	
0166	Staphylocoagulase	*coa*	0	0.14	0	0.08	-4.33	-3.91	-2.61	-3.26	0.06	-3.53	**	Yes	Yes		Yes	Extracellular	
0172	Putative zinc metalloprotease	*0172*	0	0.10			-0.67	-0.65			0.05	-0.66	*				Yes	Membrane	
0189	2-C-methyl-D-erythritol 4-phosphate cytidylyl-transferase 2	*ispD2*	0	0.17			-0.06	-0.35			0.08	-0.21						Unknown	
0210	Glycyl-glycine endopeptidase	*lytM*	0	0.03	0	-0.35	2.00	2.06	1.72	1.34	-0.08	1.78	***	Yes	Yes		Yes	Extracellular	
0249	5'-nucleotidase, lipoprotein e (P4)	*0249*	0	-0.27	0	0.22	-2.22	-2.16	-1.58	-1.75	-0.01	-1.93	***	Yes	Yes		Yes	Extracellular	
0262	Lipase 2	*geh*	0	-0.18	0	-0.19	-2.93	-2.92	-1.63	-1.75	-0.09	-2.31	**	Yes	Yes		Yes	Extracellular	
0357	30S ribosomal protein S6	*rpsF*	0	-0.14	0	-0.24	-0.36	-0.22	0.20	-0.09	-0.09	-0.12						Cytoplasm	
0371	Alkyl hydroperoxide reductase subunit F	*ahpF*	0	-0.19	0	-0.66	-0.47	-0.70	-0.86	-1.08	-0.21	-0.78	*			x		Cytoplasm	
0372	Alkyl hydroperoxide reductase subunit C	*ahpC*	0	0.13	0	-0.34	-0.05	-0.37	-0.07	-0.39	-0.05	-0.22				x	Yes	Cytoplasm	
0375	Conserved hypothetical protein	*0375*	0	-0.32			-1.06	-1.05			-0.16	-1.06					Yes	Cytoplasm	
0377	Conserved hypothetical protein	*0377*	0	0.00	0	-0.46	-0.59	-0.81	-0.50	-0.52	-0.12	-0.60	*				Yes	Cytoplasm	
0380	Inosine-5'-monophosphate dehydrogenase	*guaB*	0	-0.06	0	-0.01	0.02	-0.05	-0.40	-0.57	-0.02	-0.25				x		Cytoplasm	
0381	GMP synthase [glutamine-hydrolyzing]	*guaA*	0	-0.20			-0.31	-0.63			-0.10	-0.47				x		Cytoplasm	
0429	N-acetylmuramoyl-L-alanine amidase	*sle1*	0	-0.38	0	0.51	-1.45	-0.92	-0.12	-1.05	0.03	-0.89	*	Yes	Yes		Yes	Cell wall, non-cov.	LysM domain [6]
0443	Conserved hypothetical protein	*0443*	0	-0.07			-0.27	-0.39			-0.03	-0.33						Unknown	
0453	Methionyl-tRNA synthetase	*metS*	0	-0.33	0	-0.05	-0.87	-1.20	-0.35	-0.74	-0.09	-0.79	*					Cytoplasm	
0463	Ribose-phosphate pyrophosphokinase	*prs*	0	0.36			0.37	-0.04			0.18	0.17						Cytoplasm	
0464	50S ribosomal protein L25	*rplY*	0	0.07	0	-0.56	0.37	0.19	-0.21	-0.01	-0.12	0.08						Cytoplasm	
0472	Hypoxanthine-guanine phosphoribosyl-transferase	*hpt*	0	0.48	0	-0.65	0.11	0.44	2.31	1.36	-0.04	1.06						Cytoplasm	
0475	Cysteine synthase homolog	*cysK*	0	0.06	0	-0.08	-0.37	-0.45	-0.29	-0.44	-0.01	-0.39	***					Cytoplasm	
0479	Lysyl-tRNA synthetase	*lysS*	0	-0.25			-0.56	-0.42			-0.12	-0.49						Cytoplasm	
0481	Pyridoxal biosynthesis lyase	*pdxS*	0	-0.28	0	-1.02	-0.44	-0.39	-0.32	-0.85	-0.33	-0.50						Cytoplasm	
0482	Glutamine amidotransferase subunit	*pdxT*	0	-0.47			-0.26	-0.18			-0.23	-0.22						Cytoplasm	
0487	ATP-dependent Clp protease, ATP-binding subunit ClpC	*clpC*	0	0.40	0	-0.17	-0.05	0.02	-0.28	-0.44	0.06	-0.19				x		Cytoplasm	
0490	Glutamyl-tRNA synthetase	*gltX*	0	0.61	0	-0.54	0.01	-0.17	-0.44	-0.91	0.02	-0.38				x		Cytoplasm	
0498	Transcription antitermination protein	*nusG*	0	0.13			-0.32	-0.39			0.06	-0.35						Cytoplasm	
0499	50S ribosomal protein L11	*rplK*	0	0.15	0	-0.26	0.21	0.23	0.13	0.31	-0.03	0.22						Cytoplasm	
0500	50S ribosomal protein L1	*rplA*	0	0.23	0	-0.25	0.12	-0.16	0.06	-0.14	0.00	-0.03					Yes	Cytoplasm	
0501	50S ribosomal protein L10	*rplJ*	0	0.06	0	-0.48	0.09	-0.04	0.00	0.04	-0.10	0.02						Cytoplasm	

NWMN	Protein name	Gene	wt I	wt II	wt III	wt IV	*secDF* I	*secDF* II	*secDF* III	*secDF* IV	ø wt	ø *secDF*	*P*	SignalP [1]	Sec SP [2]	No SP(x) [2]	SecretomeP [3]	Localization [4,5]	
			fold change (log 2)																
0502	50S ribosomal protein L7/L12	*rplL*	0	0.06	0	-0.36	0.30	-0.02	-0.07	-0.12	-0.07	0.02						Cytoplasm	
0504	DNA-directed RNA polymerase subunit beta	*rpoB*	0	0.03			-0.30	-0.29			0.01	-0.30	*					Cytoplasm	
0505	DNA-directed RNA polymerase subunit beta'	*rpoC*	0	0.16	0	-0.34	-0.41	-0.49	0.07	-0.48	-0.05	-0.33						Cytoplasm	
0508	30S ribosomal protein S7	*rpsG*	0	-0.38	0	-0.70	0.42	0.36	-0.40	-0.24	-0.27	0.03						Cytoplasm	
0509	Elongation factor G	*fus*	0	0.06	0	-0.38	0.33	0.26	-0.63	-0.51	-0.08	-0.14				x		Cytoplasm	
0510	Elongation factor Tu	*tufA*	0	-0.05	0	-0.21	0.77	0.65	0.67	0.72	-0.07	0.71	***			x		Cytoplasm	
0512	Conserved hypothetical protein	*0512*	0	-0.12			-0.53	-0.89			-0.06	-0.71						Cytoplasm	
0513	Similar to chaperone protein HchA	*hchA*	0	-0.27	0	-0.44	0.05	-0.01	-0.38	-0.39	-0.18	-0.18					Yes	Cytoplasm	
0516	Probable branched-chain-amino-acid amino-transferase	*ilvE*	0	0.11	0	-0.32	0.49	0.02	-0.51	-0.65	-0.05	-0.16						Cytoplasm	
0525	Serine-aspartate repeat-containing protein E	*sdrE*	0	-0.08	0	-0.44	-0.92	-1.00	-0.81	-1.03	-0.13	-0.94	**	Yes	Pot.		Yes	Cell wall	Sortase substrate
0533	3-hexulose-6-phosphate synthase	*0533*	0	-0.32	0	-0.52	0.05	-0.16	0.37	-0.09	-0.21	0.04						Cytoplasm	
0550	Conserved hypothetical protein	*0550*	0	-0.03	0	0.56	0.62	-0.56	0.86	0.71	0.13	0.41						Unknown	
0551	Phosphate acetyltransferase	*pta*	0	0.02	0	-0.39	-0.21	-0.51	-0.53	-0.43	-0.09	-0.42	*			x		Cytoplasm	
0579	Arginyl-tRNA synthetase	*argS*	0	0.31			-1.65	-0.87			0.16	-1.26						Cytoplasm	
0588	Transcriptional regulator	*sarA*	0	0.00			-0.03	-0.26			0.00	-0.15						Cytoplasm	
0655	MarR family regulatory protein	*mgrA*	0	-0.29			0.01	-0.22			-0.14	-0.10					Yes	Cytoplasm	
0687	Glycerol phosphate lipoteichoic acid synthase	*ltaS*	0	0.04	0	-0.15	-0.70	-0.69	-0.69	-0.75	-0.03	-0.71	***				Yes	Membrane	
0721	Ribosomal protein S30EA	*0721*	0	-0.35			-0.67	-0.55			-0.18	-0.61				x		Cytoplasm	
0732	Thioredoxin reductase	*trxB*	0	0.27			-0.06	-0.15			0.14	-0.11				x		Cytoplasm	
0736	ATP-dependent Clp protease, ATP-binding subunit ClpP	*clpP*	0	-0.33			-0.36	-0.69			-0.16	-0.53				x		Cytoplasm	
0741	Glyceraldehyde-3-phosphate dehydrogenase 1	*gapA*	0	0.00	0	-0.14	0.78	0.55	0.43	0.97	-0.03	0.68	**			x		Cytoplasm	
0742	Phosphoglycerate kinase	*pgk*	0	0.05	0	0.04	0.23	0.08	-0.07	-0.39	0.02	-0.04				x		Cytoplasm	
0743	Triosephosphate isomerase	*tpi*	0	-0.18	0	-0.45	0.31	0.24	-0.36	0.19	-0.16	0.09				x		Cytoplasm	
0744	2,3-bisphosphoglycerate-independent phosphoglycerate mutase	*pgm*	0	-0.71			-3.04	-3.31			-0.35	-3.17				x		Cytoplasm	
0745	Enolase	*eno*	0	-0.13	0	-0.14	-0.01	-0.23	-0.38	-0.28	-0.07	-0.23				x		Cytoplasm	MLS
0756	Clumping factor A	*clfA*	0	0.13	0	-0.23	-1.48	-1.55	-1.34	-1.34	-0.02	-1.42	***	Yes	Yes		Yes	Cell wall	Sortase substrate
0758	Extracellular matrix protein	*emp/ssp*	0	-0.04	0	-0.36	-3.18	-3.05	-2.68	-3.00	-0.10	-2.98	***	Yes	Yes		Yes	Extracellular / CW	Can re-bind to CW, non-cov. [7]
0776	Glycine cleavage system H protein	*gcvH*	0	-0.20	0	-0.52	0.10	-0.11	-0.25	-0.42	-0.18	-0.17						Cytoplasm	
0783	CsbD-like superfamily protein	*0783*	0	-0.06	0	-0.29	-0.75	-0.87	-0.14	-0.83	-0.09	-0.65	*				Yes	Unknown	
0800	Hydrolase	*nagD*	0	0.14	0	-0.08	-0.29	-0.68	-0.33	-0.42	0.02	-0.43	**				Yes	Unknown	
0803	D-alanine-D-alanyl carrier protein ligase	*dltA*	0	-0.15			0.02	-0.24			-0.08	-0.11						Cytoplasm	
0805	D-alanyl carrier protein	*dltC*	0	-0.26	0	-0.31	0.11	0.12	0.00	0.48	-0.14	0.18						Cytoplasm	
0824	Putative peptidyl-prolyl cis-trans isomerase	*ppi*	0	-0.24	0	-0.19	0.06	-0.25	0.01	-0.38	-0.11	-0.14					Yes	Cytoplasm	
0833	Glucose-6-phosphate isomerase	*pgi*	0	-0.19	0	-0.13	-0.70	-0.51	-0.26	-0.26	-0.08	-0.43	*			x		Cytoplasm	
0836	Signal peptidase IB	*spsB*	0	-0.51			-0.77	-0.08			-0.26	-0.43					Yes	Membrane	MLS
0839	Fumarylacetacetate hydrolase family protein	*0839*	0	-0.02	0	-0.32	0.11	0.15	-0.15	-0.15	-0.09	-0.01					Yes	Cytoplasm	

NWMN	Protein name	Gene	wt I	wt II	wt III	wt IV	*secDF* I	*secDF* II	*secDF* III	*secDF* IV	ø wt	ø *secDF*	*P*	SignalP	Sec SP	No SP(x)	SecretomeP	Localization [4,5]	
			fold change (log 2)											[1]	[2]	[2]	[3]		
0845	Chaperone protein	*clpB*	0	-0.38	0	-0.01	0.20	-0.09	-2.34	-2.73	-0.10	-1.24						Cytoplasm	
0854	3-oxoacyl-[acyl-carrier-protein] synthase 2	*fabF*	0	0.00	0	-0.63	0.44	0.63	-0.27	-0.36	-0.16	0.11						Cytoplasm	
0885	2', 5' RNA ligase	*0885*	0	-0.04	0	-0.09	-0.33	-0.33	-0.44	-0.33	-0.03	-0.36	***					Cytoplasm	
0915	1,4-Dihydroxy-2-naphthoyl-CoA synthase	*memB*	0	0.22	0	-0.51	-0.18	-0.32	-0.27	-0.30	-0.07	-0.27						Cytoplasm	
0917	Cysteine protease	*sspB*	0	-0.02	0	-0.22	-1.38	-1.33	-0.65	-1.14	-0.06	-1.13	**	Yes	Yes		Yes	Extracellular	
0918	V8 protease, glutamyl endopeptidase precursor	*sspA*	0	-0.16			-1.19	-1.64			-0.08	-1.41		Yes	Yes		Yes	Extracellular	
0922	Bifunctional autolysin	*atl*	0	1.21	0	-0.34	1.67	0.91	0.19	0.12	0.22	0.72		Yes	Yes		Yes	Extracellular	
0925	Similar to cell envelope-related transcriptional attenuator	*0925*	0	-0.25	0	-0.54	-1.27	-1.31	-1.28	-1.90	-0.20	-1.44	***	Yes		x	Yes	Membrane	predicted uncleaved SP
0930	Probable quinol oxidase subunit 2	*qoxA*	0	-0.11			-0.88	-0.94			-0.06	-0.91	*				Yes	Membrane	
0932	Bifunctional protein folD	*folD*	0	-0.17			-0.40	-0.18			-0.09	-0.29						Cytoplasm	
0949	Histidine-containing phosphocarrier protein	*ptsH*	0	-0.20	0	-0.38	0.24	-0.01	-0.22	-0.15	-0.14	-0.04				x		Cytoplasm	
0950	Phosphoenolpyruvate-protein phospho-transferase	*ptsI*	0	-0.24	0	-0.46	0.04	0.06	-0.42	-0.73	-0.17	-0.26				x		Cytoplasm	
0959	Pyruvate dehydrogenase E1 component subunit alpha	*phdA*	0	-0.05	0	-0.52	0.05	-0.22	-0.29	-0.63	-0.14	-0.27				x		Cytoplasm	
0960	Pyruvate dehydrogenase E1 component subunit beta	*phdB*	0	-0.07	0	-0.28	0.20	-0.04	0.00	-0.10	-0.09	0.01				x		Cytoplasm	
0961	Dihydrolipoyllysine-residue acetyltransferase component of pyruvate dehydrogenase complex	*pdhC*	0	0.08	0	-0.35	0.02	-0.11	-0.13	-0.37	-0.07	-0.15				x		Cytoplasm	
0962	Dihydrolipoyl dehydrogenase	*pdhD*	0	-0.21	0	0.02	-0.39	-0.56	-0.24	-0.31	-0.05	-0.37	*			x		Cytoplasm	
1019	Putative major head protein Orf47	*1019*	0	0.12			-1.65	-1.53			0.06	-1.59	**					Cytoplasm	
1041	Iron-regulated surface determinant protein A	*isdA*	0	-0.08	0	-0.26	-0.53	-0.64	-0.31	-0.59	-0.08	-0.51	**	Yes	Yes		Yes	Cell wall	Sortase substrate
1057	Thioredoxin	*trxA*	0	-0.11	0	-0.35	0.79	0.56	0.18	-0.01	-0.12	0.38						Cytoplasm	
1067	FPRL1 inhibitory protein / FLIPr	*flr*	0	-0.16	0	-0.31	-4.07	-3.87	-3.04	-3.25	-0.12	-3.56	***	Yes	Yes		Yes	Extracellular	
1069	Fibrinogen-binding protein	*efb/fib*	0	0.27	0	0.09	-1.49	-1.11	-1.38	-1.34	0.09	-1.33	***	Yes	Yes		Yes	Extracellular / CW	Can re-bind to CW, non-cov. [7]
1073	Alpha-hemolysin	*hla*	0	0.02	0	-0.46	-1.16	-1.44	-0.67	-0.97	-0.11	-1.06	**	Yes	Yes		Yes	Extracellular	
1086	Uncharacterized N-acetyltransferase	*1086*	0	0.07			-0.68	-0.54			0.04	-0.61	*					Unknown	
1096	Cell division protein	*ftsZ*	0	-0.19	0	-0.60	-0.29	-0.16	-0.47	-0.24	-0.20	-0.29						Cytoplasm	
1103	Isoleucyl-tRNA synthetase OS	*ileS*	0	0.08	0	-0.19	-0.45	-0.44	-0.12	-0.51	-0.03	-0.38	*			x		Cytoplasm	
1125	Peptide deformylase	*def*	0	-0.60			-0.34	-0.79			-0.30	-0.57						Cytoplasm	
1139	Phosphate acyltransferase	*plsX*	0	1.27			-0.86	0.15			0.63	-0.36						Cytoplasm	
1140	Malonyl CoA-acyl carrier protein transacylase	*fabD*	0	-0.63			-0.15	-0.77			-0.31	-0.46						Cytoplasm	
1141	3-oxoacyl-[acyl-carrier-protein] reductase	*fabG*	0	-0.40	0	0.12	-0.65	-0.83	-0.47	-0.32	-0.07	-0.57	*					Cytoplasm	
1148	30S ribosomal protein S16	*rpsP*	0	-0.01	0	-0.39	0.13	-0.14	-0.42	-0.44	-0.10	-0.22					Yes	Cytoplasm	
1155	Succinyl-CoA ligase [ADP-forming] subunit β	*sucC*	0	0.02	0	-0.38	-0.40	-0.59	-0.56	-0.85	-0.09	-0.60	**					Cytoplasm	
1156	Succinyl-CoA ligase [ADP-forming] subunit α	*sucD*	0	0.27	0	-0.12	-0.35	-0.49	0.12	-0.48	0.04	-0.30				x		Cytoplasm	
1165	GTP-sensing transcriptional pleiotropic repressor	*codY*	0	-0.13	0	0.19	0.08	-0.11	0.53	0.86	0.02	0.34						Cytoplasm	

NWMN	Protein name	Gene	wt I	wt II	wt III	wt IV	*secDF* I	*secDF* II	*secDF* III	*secDF* IV	ø wt	ø *secDF*	*P*	SignalP	Sec SP	No SP(x)	SecretomeP	Localization [4,5]	
			fold change (log 2)											[1]	[2]	[2]	[3]		
1166	30S ribosomal protein S2	*rpsB*	0	-0.47	0	-0.22	-0.41	-0.40	-0.48	-0.62	-0.17	-0.48						Cytoplasm	
1167	Elongation factor Ts	*tsf*	0	-0.12	0	-0.45	0.08	-0.07	-0.06	-0.13	-0.14	-0.05				x		Cytoplasm	
1169	Ribosome-recycling factor	*frr*	0	0.06	0	-0.44	0.02	-0.22	0.28	0.04	-0.09	0.03						Cytoplasm	
1178	Translation initiation factor IF-2	*infB*	0	-0.23			-0.07	0.11			-0.11	0.02						Cytoplasm	
1181	Polyribonucleotide nucleotidyltransferase	*ribF*	0	-0.44			-1.25	-1.02			-0.22	-1.13						Cytoplasm	
1182	30S ribosomal protein S15	*rpsO*	0	-0.28			-0.39	-0.50			-0.14	-0.44						Cytoplasm	
1208	Glycerol kinase	*glpK*	0	0.19			-0.58	-0.55			0.09	-0.57						Cytoplasm	
1217	Glutamine synthetase	*glnA*	0	-0.03	0	0.08	0.32	0.16	0.59	0.45	0.01	0.38	*			x		Cytoplasm	
1236	Thermonuclease	*nuc*	0	0.00	0	-0.21	-0.57	-0.63	-0.33	-0.66	-0.05	-0.55	**				Yes	Extracellular	
1246	Catalase	*katA*	0	-0.04	0	-0.42	-0.57	-0.75	-0.79	-0.81	-0.11	-0.73	**			x	Yes	Cytoplasm	
1254	Transketolase	*tkt*	0	-0.12	0	-0.13	-0.13	-0.17	-0.07	-0.26	-0.06	-0.16				x	Yes	Unknown	
1263	Aconitate hydratase	*acnA*	0	-0.21	0	-0.11	-0.25	-0.46	-0.39	-0.19	-0.08	-0.32	*				Yes	Cytoplasm	
1274	Regulatory protein	*msrR*	0	-0.02	0	-0.19	-1.46	-1.00	-1.04	-1.14	-0.05	-1.16	***				Yes	Membrane	
1275	4-oxalocrotonate tautomerase	*1275*	0	0.12			0.01	-0.07			0.06	-0.03						Cytoplasm	
1313	Cold shock protein	*cspA*	0	-0.20	0	-0.44	0.44	0.35	0.62	0.12	-0.16	0.38	*			x	Yes	Cytoplasm	
1332	Probable CtpA-like serine protease	*1332*	0	-0.48			-1.22	-0.89			-0.24	-1.05					Yes	Membrane	
1333	Phosphotransferase system glucose-specific IIA component	*crr*	0	-0.46			-0.01	-0.32			-0.23	-0.17						Cytoplasm	
1339	Conserved hypothetical protein	*1339*	0	0.32			-0.36	-0.46			0.16	-0.41						Cytoplasm	
1365	Asparaginyl-tRNA synthetase	*asnS*	0	-0.10			-0.55	-0.59			-0.05	-0.57	*					Cytoplasm	
1378	Nucleoside diphosphate kinase	*ndk*	0	-0.05			-0.53	-0.61			-0.02	-0.57	*					Cytoplasm	
1382	DNA-binding protein HU	*hup*	0	0.10	0	-0.38	0.48	0.24	0.48	0.39	-0.07	0.40	*					Cytoplasm	
1385	30S ribosomal protein S1	*rpsA*	0	-0.09	0	-0.37	-0.42	-0.55	-0.15	-0.33	-0.11	-0.36				x		Cytoplasm	
1386	Cytidylate kinase	*cmk*	0	0.34			0.86	0.17			0.17	0.52						Cytoplasm	
1389	Elastin-binding protein	*ebpS*			0	-0.50			-1.13	-1.23	-0.25	-1.18				x	Yes	Membrane	MLS
1417	6-phosphogluconate dehydrogenase, decarboxylating	*gnd*	0	0.00			0.24	-0.10			0.00	0.07				x		Cytoplasm	
1456	Superoxide dismutase [Mn/Fe]	*sodA*	0	0.26	0	-0.48	-0.26	-0.24	-0.21	-0.43	-0.06	-0.28				x	Yes	Extracellular	No SP
1468	Glycyl-tRNA synthetase	*glyS*	0	0.07	0	-0.07	0.30	0.06	-0.14	-0.08	0.00	0.04						Cytoplasm	
1483	Chaperone protein	*dnaK*	0	0.02	0	-0.40	0.12	0.02	0.14	0.07	-0.10	0.09				x	Yes	Cytoplasm	
1484	Hsp-70 cofactor	*grpE*	0	0.08	0	-0.43	-0.51	-0.57	-0.09	-0.35	-0.09	-0.38				x	Yes	Cytoplasm	
1488	30S ribosomal protein S20	*rpsT*	0	-0.37			-0.59	-0.95			-0.19	-0.77					Yes	Cytoplasm	
1516	Conserved hypothetical protein	*1516*	0	0.00			0.14	-0.02			0.00	0.06						Cytoplasm	
1547	50S ribosomal protein L27	*rpmA*	0	0.14	0	-0.53	-0.02	-0.10	-0.42	-0.47	-0.10	-0.25					Yes	Cytoplasm	
1549	50S ribosomal protein L21	*rplU*	0	0.14	0	-0.97	-0.77	-0.36	-0.55	-0.92	-0.21	-0.65						Cytoplasm	
1558	Valyl-tRNA synthetase	*valS*	0	0.35			0.19	-0.13			0.18	0.03						Cytoplasm	
1569	Trigger factor	*tig*	0	-0.13	0	-0.21	-0.03	-0.23	-0.40	-0.40	-0.09	-0.26				x		Cytoplasm	
1574	Translation initiation factor IF-3	*infC*	0	-0.23			-1.13	-0.50			-0.12	-0.81						Cytoplasm	
1576	Threonyl-tRNA synthetase	*thrS*	0	0.19			0.03	-0.48			0.09	-0.23						Cytoplasm	
1587	Isocitrate dehydrogenase [NADP]	*citC*	0	-0.11	0	-0.35	-0.51	-0.71	-0.49	-1.00	-0.11	-0.68	**					Cytoplasm	

NWMN	Protein name	Gene	wt I	wt II	wt III	wt IV	*secDF* I	*secDF* II	*secDF* III	*secDF* IV	ø wt	ø *secDF*	*P*	SignalP	Sec SP	No SP(x)	SecretomeP	Localization [4,5]	
			fold change (log 2)											[1]	[2]	[2]	[3]		
1592	Pyruvate kinase	*pyk*	0	-0.32			-0.72	-0.60			-0.16	-0.66						Cytoplasm	
1593	6-phosphofructokinase	*pfk*	0	-0.14	0	-0.35	-0.56	-0.23	-0.40	-0.24	-0.12	-0.36						Cytoplasm	
1601	Metal-dependent hydrolase	*1601*	0	0.93			0.81	-0.03			0.46	0.39						Cytoplasm	
1604	Universal stress protein family protein	*1604*	0	-0.07	0	-0.40	-0.19	-0.25	-0.43	-0.31	-0.12	-0.29				x		Cytoplasm	
1609	Probable thiol peroxidase	*thiI*	0	-0.04	0	-0.09	-0.25	-0.23	0.24	-0.32	-0.03	-0.14						Cytoplasm	
1613	30S ribosomal protein S4	*rpsd*	0	0.13			0.05	-0.09			0.07	-0.02						Cytoplasm	
1625	Formate--tetrahydrofolate ligase	*fhs*	0	0.25	0	-0.39	-0.21	-0.25	-0.18	-0.45	-0.04	-0.27				x		Cytoplasm	
1643	D-alanine aminotransferase	*dat*	0	-0.28			0.29	0.44			-0.14	0.36						Cytoplasm	
1681	Phosphoenolpyruvate carboxykinase [ATP]	*pckA*	0	-0.11			-0.75	-0.85			-0.05	-0.80	**				Yes	Cytoplasm	
1726	Signal transduction protein TRAP (RNAIII-activating protein TRAP)	*traP*	0	-0.08			-0.18	-0.56			-0.04	-0.37				x	Yes	Cytoplasm	
1733	Foldase protein	*prsA*	0	0.01	0	-0.31	-0.49	-0.49	-0.66	-0.82	-0.08	-0.62	**	Yes			Yes	Lipoprotein	
1737	Conserved hypothetical protein	*1737*	0	0.23	0	-0.40	0.83	0.71	-0.14	-0.03	-0.04	0.34						Cytoplasm	
1767	ThiJ/PfpI family protein	*1767*	0	-0.19	0	-0.58	-0.36	-0.68	-0.56	-0.71	-0.19	-0.58						Cytoplasm	
1831	Ferritin	*ftnA*	0	0.31	0	-0.25	-0.20	-0.49	-0.36	-0.22	0.01	-0.31						Cytoplasm	
1837	Aspartyl/glutamyl-tRNA(Asn/Gln) amidotransferase subunit B	*gatB*	0	-0.37	0	-0.74	-0.23	-0.56	-0.05	-0.24	-0.28	-0.27						Cytoplasm	
1838	Glutamyl-tRNA(Gln) amidotransferase subunit A	*gatA*	0	0.16			0.62	0.34			0.08	0.48					Yes	Cytoplasm	
1839	Aspartyl/glutamyl-tRNA(Asn/Gln) amidotransferase subunit C	*gatC*	0	0.23	0	0.03	-0.40	-0.51	-0.55	-0.62	0.06	-0.52	***				Yes	Cytoplasm	
1847	Staphopain thiol protease	*scpA*	0	-0.23			-2.14	-2.22			-0.12	-2.18	*	Yes	Yes		Yes	Extracellular	
1857	Probable manganese-dependent inorganic pyrophosphatase	*ppaC*	0	-0.07	0	-0.03	-1.00	-0.38	-2.13	-2.27	-0.03	-1.45						Cytoplasm	
1859	Conserved hypothetical protein	*1859*	0	-0.77			-0.91	-1.13			-0.38	-1.02				x		Cytoplasm	
1872	65 kDa membrane protein	*eap/map*	0	-0.42	0	-0.27	-1.79	-1.61	-2.06	-2.21	-0.17	-1.92	***	Yes	Yes		Yes	Extracellular / CW	Can re-bind to CW, non-cov. [8]
1876	Staphylococcal complement inhibitor	*scn*	0	0.20	0	-0.05	-1.65	-1.66	-3.18	-3.46	0.04	-2.49	*	Yes	Yes		Yes	Extracellular	
1877	Chemotaxis inhibitory protein	*chp*	0	0.47	0	0.63	-2.52	-2.29	-1.25	-1.62	0.28	-1.92	**	Yes	Yes		Yes	Extracellular	
1880	Staphylokinase	*sak*	0	0.08	0	-0.48	-0.87	-0.87	-0.79	-0.82	-0.10	-0.84	**	Yes	Yes		Yes	Extracellular	
1883	Enterotoxin type A	*sea*	0	0.05	0	-0.08	-1.95	-1.89	-1.29	-1.40	-0.01	-1.63	**	Yes			Yes	Extracellular	
1927	Leukocidin/hemolysin toxin family F subunit	*lukF*	0	0.12	0	-0.21	-0.42	-0.54	-1.09	-0.87	-0.02	-0.73	*	Yes	Yes		Yes	Extracellular	
1928	Leukocidin/hemolysin toxin family S subunit	*lukS*	0	-0.03	0	-0.31	-1.42	-1.70	-1.16	-1.14	-0.09	-1.36	***	Yes	Yes		Yes	Extracellular	
1937	Chaperonin 60 kDa sunbunit	*groEL*	0	0.03	0	-0.61	-0.28	-0.51	-0.41	-0.47	-0.15	-0.42				x		Cytoplasm	
1938	10 kDa chaperonin	*groES*			0	-0.79			-0.41	-0.69	-0.40	-0.55				x		Cytoplasm	
1972	Anti-sigma-B factor antagonist	*rsbV*	0	-0.15	0	-0.11	-0.79	-0.87	-0.29	-0.09	-0.07	-0.51						Cytoplasm	
1999	Probable transglycosylase	*sceD*	0	0.09	0	-0.49	1.81	1.81	1.60	1.38	-0.10	1.65	***	Yes	Yes		Yes	Extracellular	Can ionically bind to cell wall [9]
2004	UDP-N-acetylglucosamine 1-carboxyvinyltransferase 1	*murA1*	0	1.01			-1.90	-0.17			0.51	-1.03						Cytoplasm	
2007	ATP synthase subunit beta	*atpD*	0	-0.18			-0.47	-0.74			-0.09	-0.60						Cytoplasm	

NWMN	Protein name	Gene	wt I	wt II	wt III	wt IV	*secDF* I	*secDF* II	*secDF* III	*secDF* IV	ø wt	ø *secDF*	*P*	SignalP	Sec SP	No SP(x)	SecretomeP	Localization [4,5]	
			fold change (log 2)											[1]	[2]	[2]	[3]		
2009	ATP synthase subunit alpha	*atpA*	0	-0.08			0.08	-0.30			-0.04	-0.11						Cytoplasm	
2016	Uracil phosphoribosyltransferase	*upp*	0	-0.41			-0.09	-0.14			-0.21	-0.11						Cytoplasm	
2024	50S ribosomal protein L31 type B	*rpmE*	0	-0.08	0	-0.36	-0.25	-0.48	-0.25	-0.58	-0.11	-0.39					Yes	Cytoplasm	
2026	Putative aldehyde dehydrogenase	*2026*	0	-0.21			-0.29	-0.55			-0.11	-0.42						Cytoplasm	
2029	Fructose-bisphosphate aldolase	*fbaA*	0	-0.23	0	-0.24	0.10	-0.06	-0.27	-0.18	-0.12	-0.10				x		Cytoplasm	
2031	CTP synthase	*pyrG*	0	0.12			-0.28	0.52			0.06	0.12						Cytoplasm	
2032	Probable DNA-directed RNA polymerase subunit delta	*rpoE*	0	-0.11			-0.06	-0.33			-0.06	-0.20					Yes	Cytoplasm	
2041	Deoxyribose-phosphate aldolase 1	*deoC*	0	-0.43			-0.63	-0.47			-0.21	-0.55						Cytoplasm	
2056	Glucosamine--fructose-6-phosphate aminotransferase [isomerizing]	*glmS*	0	0.04			0.51	-0.30			0.02	0.11						Cytoplasm	
2062	Phosphoglucosamine mutase	*glmM*	0	0.10			0.07	-0.11			0.05	-0.02						Cytoplasm	
2074	Conserved hypothetical protein	*2074*	0	0.13			-0.33	-0.36			0.06	-0.34				x	Yes	Unknown	
2086	Alkaline shock protein 23	*asp23*	0	0.06	0	-0.18	-0.55	-0.97	-0.65	-0.50	-0.03	-0.67	**			x	Yes	Unknown	
2119	30S ribosomal protein S9	*rpsI*			0	-0.66			-0.30	-0.95	-0.33	-0.63						Cytoplasm	
2120	50S ribosomal protein L13	*rplM*	0	-0.06	0	-0.51	-0.33	-0.41	-0.49	-0.56	-0.14	-0.45				x	Yes	Cytoplasm	
2125	50S ribosomal protein L17	*rplQ*	0	0.02	0	-0.24	0.04	-0.06	-0.19	-0.34	-0.06	-0.14						Cytoplasm	
2126	DNA-directed RNA polymerase subunit alpha	*rpoA*	0	-0.02			-0.14	-0.06			-0.01	-0.10						Cytoplasm	
2127	30S ribosomal protein S11	*rpsK*	0	0.11			-0.23	-0.29			0.06	-0.26					Yes	Cytoplasm	
2128	30S ribosomal protein S13	*rpsM*			0	-0.06			0.36	0.54	-0.03	0.45						Cytoplasm	
2130	Translation initiation factor IF-1	*infA*	0	-0.64	0	-0.10	0.52	0.35	0.88	0.88	-0.18	0.66	**					Cytoplasm	
2131	Adenylate kinase	*adk*	0	-0.02	0	-0.31	0.24	0.05	-0.06	-0.33	-0.08	-0.03						Cytoplasm	
2133	50S ribosomal protein L15	*rplO*	0	-0.01	0	-0.40	-0.29	-0.37	-1.00	-0.81	-0.10	-0.62	*					Cytoplasm	
2134	50S ribosomal protein L30	*rpmD*	0	0.02			-0.02	-0.13			0.01	-0.08					Yes	Cytoplasm	
2135	30S ribosomal protein S5	*rpsE*	0	0.02	0	-0.77	-0.07	-0.23	0.27	0.01	-0.19	-0.01						Cytoplasm	
2137	50S ribosomal protein L6	*rplF*	0	0.00	0	-0.29	0.19	0.13	0.09	0.17	-0.07	0.15					Yes	Cytoplasm	
2138	30S ribosomal protein S8	*rpsH*	0	0.22	0	-0.62	-0.03	0.09	-0.26	-0.20	-0.10	-0.10						Cytoplasm	
2140	50S ribosomal protein L5	*rplE*	0	0.05	0	-0.53	-0.04	-0.05	-0.42	-0.05	-0.12	-0.14						Cytoplasm	
2141	50S ribosomal protein L24	*rplX*	0	0.20			0.27	0.09			0.10	0.18					Yes	Cytoplasm	
2143	30S ribosomal protein S17	*rpsQ*	0	0.07			0.05	0.14			0.04	0.09					Yes	Cytoplasm	
2144	50S ribosomal protein L29	*rpmC*	0	0.05	0	-0.44	-0.25	-0.24	0.07	-0.08	-0.10	-0.13						Cytoplasm	
2145	50S ribosomal protein L16	*rplP*	0	-0.51			-0.98	-0.79			-0.25	-0.88						Cytoplasm	
2146	30S ribosomal protein S3	*rpsC*	0	0.08			0.05	-0.15			0.04	-0.05						Cytoplasm	
2147	50S ribosomal protein L22	*rplV*	0	0.13	0	-0.65	-0.73	-0.40	-0.28	-0.42	-0.13	-0.46						Cytoplasm	
2148	30S ribosomal protein S19	*rpsS*	0	0.13	0	-0.28	-0.46	-0.48	-0.26	-0.53	-0.04	-0.43	*					Cytoplasm	
2149	50S ribosomal protein L2	*rplB*	0	-0.19	0	-0.21	0.05	-0.05	-0.22	-0.31	-0.10	-0.13						Cytoplasm	
2150	50S ribosomal protein L23	*rplW*	0	0.21			0.66	0.42			0.11	0.54						Cytoplasm	
2151	50S ribosomal protein L4	*rplD*	0	-0.93			-0.60	-1.20			-0.47	-0.90						Cytoplasm	
2199	Staphylococcal secretory antigen	*ssaA*	0	0.05	0	-0.06	-1.95	-1.80	-1.75	-1.55	0.00	-1.76	***	Yes	Yes		Yes	Extracellular	Can ionically bind to cell wall [9]

NWMN	Protein name	Gene	wt I	wt II	wt III	wt IV	*secDF* I	*secDF* II	*secDF* III	*secDF* IV	ø wt	ø *secDF*	*P*	SignalP [1]	Sec SP [2]	No SP(x) [2]	SecretomeP [3]	Localization [4,5]	
			fold change (log 2)																
2205	Putative 2-hydroxyacid dehydrogenase	*2205*	0	-0.50	0	-0.60	-0.12	-0.43	-0.25	-0.66	-0.27	-0.36						Cytoplasm	
2270	Conserved hypothetical protein	*2270*	0	-0.24	0	0.04	-0.47	-1.03	-0.65	-1.06	-0.05	-0.80	**	Yes			Yes	Lipoprotein	MLS
2317	Immunoglobulin-binding protein	*sbi*	0	-0.03	0	-0.16	-1.33	-1.23	-1.20	-1.22	-0.05	-1.25	***	Yes	Yes		Yes	Extracellular	
2318	Gamma-hemolysin component A	*hlgA*	0	-0.03	0	-0.33	-0.09	-0.23	-0.67	-0.87	-0.09	-0.46		Yes	Yes		Yes	Extracellular	
2319	Gamma-hemolysin component C	*hlgC*	0	0.08	0	-0.26	-0.32	-0.88	-1.00	-0.99	-0.04	-0.79	*	Yes	Yes		Yes	Extracellular	
2320	Gamma-hemolysin component B	*hlgB*	0	-0.20	0	-0.23	-0.30	-0.58	-0.50	-0.78	-0.11	-0.54	*	Yes	Yes		Yes	Extracellular	
2399	Fibronectin-binding protein A	*fnbA*	0	-0.08	0	-0.07	-2.01	-1.92	-2.78	-2.78	-0.04	-2.37	**	Yes	Yes		Yes	Cell wall	Sortase substrate[1]
2448	ATP-dependent Clp protease, ATP-binding subunit	*clpL*	0	0.11			-0.71	-0.88			0.05	-0.80	*					Cytoplasm	MLS
2454	1-pyrroline-5-carboxylate dehydrogenase	*rocA*	0	-0.21	0	-0.58	-0.67	-0.81	-0.76	-0.84	-0.20	-0.77	*			x		Cytoplasm	
2467	O-acetyltransferase	*oatA*	0	0.35	0	-0.23	-1.07	-1.00	-1.13	-1.32	0.03	-1.13	***				Yes	Membrane	
2469	Probable transglycosylase	*isaA*	0	-0.11	0	-0.04	-1.83	-1.87	-1.43	-1.35	-0.04	-1.62	**	Yes	Yes		Yes	Extracellular	Can ionically bind to cell wall [9]
2480	Alpha/beta hydrolase fold family protein	*2480*	0	-0.04			-0.52	-0.22			-0.02	-0.37					Yes	Cytoplasm	
2503	Fructose-bisphosphate aldolase class 1	*fda*	0	-0.03	0	-0.39	0.37	0.49	0.24	0.10	-0.10	0.30	*			x		Cytoplasm	
2504	Probable malate:quinone oxidoreductase 2	*mqo2*	0	0.31			-0.52	-0.54			0.15	-0.53						Cytoplasm	MLS
2529	Clumping factor B	*clfB*	0	-0.17	0	-0.30	-2.58	-2.64	-1.55	-1.80	-0.12	-2.15	**	yes	Yes		yes	Cell wall	Sortase substrate
2537	Immunodominant staphylococcal antigen B	*isaB*	0	-0.05	0	-0.07	-0.60	-0.56	-1.09	-1.40	-0.03	-0.91	*	yes	Yes		yes	Extracellular	
2569	Lipase 1	*lip*	0	0.27	0	-0.15	-0.68	-0.80	-0.54	-0.69	0.03	-0.67	**	yes	Yes		yes	Extracellular	
2624	Delta-hemolysin	*hld*	0	-0.78	0	-1.09	-2.17	-2.12	-1.95	-2.12	-0.47	-2.09	**					Extracellular	No SP

(x) Extracellular, without known signal peptide; MLS, multiple localization sites; [1], FnBPA is truncated due to a point mutation leading to a stop codon. Thus it lacks the sortase motif LPXTG and is entirely secreted [10]; *, $P < 0.05$; **, $P < 0.01$; *** $P < 0.001$.

1. Petersen TN, Brunak S, von Heijne G, Nielsen H (2011) SignalP 4.0: discriminating signal peptides from transmembrane regions. Nat Methods 8: 785-786.
2. Sibbald MJJB, Ziebandt AK, Engelmann S, Hecker M, de Jong A, et al. (2006) Mapping the pathways to staphylococcal pathogenesis by comparative secretomics. Microbiol Mol Biol Rev 70: 755-788.
3. Bendtsen J, Kiemer L, Fausboll A, Brunak S (2005) Non-classical protein secretion in bacteria. BMC Microbiol 5: 58.
4. Yu NY, Wagner JR, Laird MR, Melli G, Rey S, et al. (2010) PSORTb 3.0: improved protein subcellular localization prediction with refined localization subcategories and predictive capabilities for all prokaryotes. Bioinformatics 26: 1608-1615.
5. Krogh A, Larsson Br, von Heijne G, Sonnhammer ELL (2001) Predicting transmembrane protein topology with a hidden markov model: application to complete genomes. J Mol Biol 305: 567-580.
6. Buist G, Steen A, Kok J, Kuipers OP (2008) LysM, a widely distributed protein motif for binding to (peptido)glycans. Mol Microbiol 68: 838-847.
7. Chavakis T, Wiechmann K, Preissner KT, Herrmann M (2005) *Staphylococcus aureus* interactions with the endothelium: the role of bacterial "secretable expanded repertoire adhesive molecules" (SERAM) in disturbing host defense systems. Thromb Haemost 94: 278-285.
8. Palma M, Haggar A, Flock J-I (1999) Adherence of *Staphylococcus aureus* is enhanced by an endogenous secreted protein with broad binding activity. J Bacteriol 181: 2840-2845.
9. Stapleton MR, Horsburgh MJ, Hayhurst EJ, Wright L, Jonsson I-M, et al. (2007) Characterization of IsaA and SceD, two putative lytic transglycosylases of *Staphylococcus aureus*. J Bacteriol 189: 7316-7325.
10. Grundmeier M, Hussain M, Becker P, Heilmann C, Peters G, et al. (2004) Truncation of fibronectin-binding proteins in *Staphylococcus aureus* strain Newman leads to deficient adherence and host cell invasion due to loss of the cell wall anchor function. Infect Immun 72: 7155-7163.
